# Population Scale Analysis of Centromeric Satellite DNA Reveals Highly Dynamic Evolutionary Patterns and Genomic Organization in Long-Tailed and Rhesus Macaques

**DOI:** 10.3390/cells11121953

**Published:** 2022-06-17

**Authors:** Worapong Singchat, Syed Farhan Ahmad, Kitipong Jaisamut, Thitipong Panthum, Nattakan Ariyaraphong, Ekaphan Kraichak, Narongrit Muangmai, Prateep Duengkae, Sunchai Payungporn, Suchinda Malaivijitnond, Kornsorn Srikulnath

**Affiliations:** 1 Animal Genomics and Bioresource Research Unit (AGB Research Unit), Faculty of Science, Kasetsart University, Bangkok 10900, Thailand; worapong.si@ku.th (W.S.); syedfarhan.a@ku.th (S.F.A.); kjaisamut10@gmail.com (K.J.); thitipong.pa@ku.th (T.P.); nattakan.ari@ku.th (N.A.); ekaphan.k@ku.th (E.K.); ffisnrm@ku.ac.th (N.M.); prateep.du@ku.ac.th (P.D.); suchinda.m@chula.ac.th (S.M.); 2 Special Research Unit for Wildlife Genomics (SRUWG), Department of Forest Biology, Faculty of Forestry, Kasetsart University, 50 Ngamwongwan, Chatuchak, Bangkok 10900, Thailand; 3 The International Undergraduate Program in Bioscience and Technology, Faculty of Science, Kasetsart University, 50 Ngamwongwan, Chatuchak, Bangkok 10900, Thailand; 4 Department of Botany, Faculty of Science, Kasetsart University, Bangkok 10900, Thailand; 5 Department of Fishery Biology, Faculty of Fisheries, Kasetsart University, Bangkok 10900, Thailand; 6 Department of Biochemistry, Faculty of Medicine, Chulalongkorn University, Bangkok 10330, Thailand; sp.medbiochemcu@gmail.com; 7 Department of Biology, Faculty of Science, Chulalongkorn University, Bangkok 10330, Thailand; 8 National Primate Research Center of Thailand-Chulalongkorn University, Saraburi 18110, Thailand; 9 Laboratory of Animal Cytogenetics and Comparative Genomics (ACCG), Department of Genetics, Faculty of Science, Kasetsart University, Bangkok 10900, Thailand; 10 Amphibian Research Center, Hiroshima University, 1-3-1 Kagamiyama, Higashihiroshima 739-8526, Japan

**Keywords:** centromere, diversity, satellite DNA, macaque, primate

## Abstract

Centromeric satellite DNA (cen-satDNA) consists of highly divergent repeat monomers, each approximately 171 base pairs in length. Here, we investigated the genetic diversity in the centromeric region of two primate species: long-tailed (*Macaca fascicularis*) and rhesus (*Macaca mulatta*) macaques. Fluorescence in situ hybridization and bioinformatic analysis showed the chromosome-specific organization and dynamic nature of cen-satDNAsequences, and their substantial diversity, with distinct subfamilies across macaque populations, suggesting increased turnovers. Comparative genomics identified high level polymorphisms spanning a 120 bp deletion region and a remarkable interspecific variability in cen-satDNA size and structure. Population structure analysis detected admixture patterns within populations, indicating their high divergence and rapid evolution. However, differences in cen-satDNA profiles appear to not be involved in hybrid incompatibility between the two species. Our study provides a genomic landscape of centromeric repeats in wild macaques and opens new avenues for exploring their impact on the adaptive evolution and speciation of primates.

## 1. Introduction

Centromeres are essential chromatin domains for chromosome segregation during cell division and for the maintenance of genome stability across eukaryotes [1,2]. Centromeric DNA is mainly composed of tandem arrays of repetitive DNA sequences with multiple repeat units known as satellite DNA (satDNA) [3,4]. Owing to this satellite-rich architecture, centromeres tend to have high rates of structural mutations that occur through replication slippage, unequal crossing over, and transposition [5,6,7,8,9]. These processes actively contribute to variability in the repeat size, number, and nucleotide sequences between species or populations [7,8,9,10,11,12], and also result in the appearance of epigenetic modifications in centromeric satDNA (cen-satDNA), affecting centromeric functions [5,13,14,15,16,17]. Within a species, many satDNA copies have been shown to undergo a process known as molecular drive that homogenizes the sequence within the genome, subsequently fixing it in the sexual population [18,19,20]. Several mechanisms have been postulated for this homogenization, including gene conversion and unequal crossing over [7,21,22]. Multiple satDNA copies exhibit higher similarity within a species, termed familial satDNA, as opposed to within the same satDNA sequence pattern of related species in concerted evolution. Different satDNA variants might exist within the genome of a species as a consequence of satDNA turnover mechanisms, leading to the emergence of new specific satDNA families. Two families located in the same genome are highly similar; they maintain the integrity of each specific individual family and exhibit high homogeneity of repetitive sequences [7,8,9,11,12,23]. Following “the library model”, different satDNA families can efficiently change the arrangement of DNA sequences in heterochromatin by replacing one dominant satDNA family with another that is less well represented and differs in nucleotide sequences or copy numbers in related species [24]. Natural selection could drive the biased amplification of one satDNA family more than others [17,18,25]. Varieties among satDNA families are attributed to mutation rate, species, chromosome morphology, population size, and reproductive mode. Interestingly, satDNA varieties can exist within a single satDNA family, known as subfamilies, with each one being represented by a specific repetitive sequence.

Cen-satDNA sequences, which occupy a substantial portion of primate centromeres, are mainly constituted by “alpha-satellite” arrays [26,27,28,29,30]. Alpha-satDNA variants have been associated with differences in the stability of kinetochore protein binding, affecting the fidelity of chromosome segregation [31,32]. The organization and unit of periodicity of these arrays are specific to each primate species or chromosome [33,34]. Primate species comparison of alpha-satDNA has revealed that human alpha-satDNA shares 80–86% identity with that of chimpanzees (*Pan troglodytes*, Blumenbach, 1775) [35], western gorillas (*Gorilla gorilla*, Savage, 1847) [36], and Sumatran orangutans (*Pongo abelii*, Lesson, 1827) [37], indicating the presence of alpha-satDNA in a common primate ancestor [22,38,39]. The structure and content of alpha-satDNA in primates have changed rapidly over relatively short periods of evolutionary time [22,39,40]. Whereas variability in cen-satDNA has been shown to have a pivotal effect on phenotypic constraints in vertebrates such as mice, with the bias of non-Mendelian chromosome transmission in heterozygotes, known as centromere drive in the population [41,42]. An improved knowledge of cen-satDNA diversity is a crucial first step toward understanding the potential phenotypic consequences of variation in species lineages.

Rhesus macaques (*Macaca mulatta*, Zimmermann, 1780) [43] and long-tailed macaques (Macaca fascicularis, Raffles, 1821 [44] are evolutionarily successful and intensively studied nonhuman primates (NHPs) [45,46]. These species have the largest natural geographic range of any NHPs, extending from India in the west across Asia to the Pacific coast of China and south into Vietnam and Thailand, exhibiting their outstanding ecological flexibility and adaptability [46,47]. Their adaptability and overall physiological characters are similar to those of humans; therefore, these macaques are widely used as animal models for drug and vaccine testing related to human health and disease [46,48]. However, hybridization and introgression have been observed between the two macaque species in Laos, Thailand, and Vietnam [46,49,50,51]. The gradient genomic profiles of the two macaque species show varying degrees of hybridization according to their geographic distribution, leading to likely variability in phenotypic and physiological aspects such as a susceptibility to pathogens [46,52,53,54,55]. This complicates the genetic variability among different populations; hence, close attention is needed to elucidate the genetic components of macaques. Based on our preliminary genomic analysis, the comparison of cen-satDNA between the rhesus macaque and human genomes revealed differences of 6–10%, suggesting the existence of alpha-satDNA in a common primate ancestor [9]. However, the phenomenon of cen-satDNA with variable phenotypic consequences and diversity is highly complex between species or populations, as observed in other lineages [12,29,56,57,58,59]. This leads us to predict that cen-satDNA might affect the populations of the two macaque species according to their geographic distributions and gradient genomic profiles. An examination of the organization and diversity of cen-satDNA within populations of these two species is thus necessary to understand the mechanism by which satDNA affects species diversity and population divergence in both long-tailed and rhesus macaques. To better understand the initial steps of cen-satDNA divergence, we characterized the organization and sequence divergence of cen-satDNA in geographically distinct populations of the two macaque species. We hypothesized that (i) the organization and diversity of cen-satDNA in the two macaque genomes (long-tailed and rhesus macaques) might have followed independent evolution, and that (ii) cen-satDNA sequences might show population/species level divergence. To test our hypotheses, we isolated cen-satDNA from long-tailed macaques and sequenced specific cen-satDNA of long-tailed and rhesus macaques sampled from 18 populations in Thailand [60]. We also examined the genomic organization and variation in the cen-satDNA sequence composition across a panel of diverse macaque species. Our study provides the groundwork for future functional studies on the consequences of natural genetic variations in essential chromatin domains.

## 2. Materials and Methods

### 2.1. Specimen Collection and DNA Extraction

**Figure 1:**
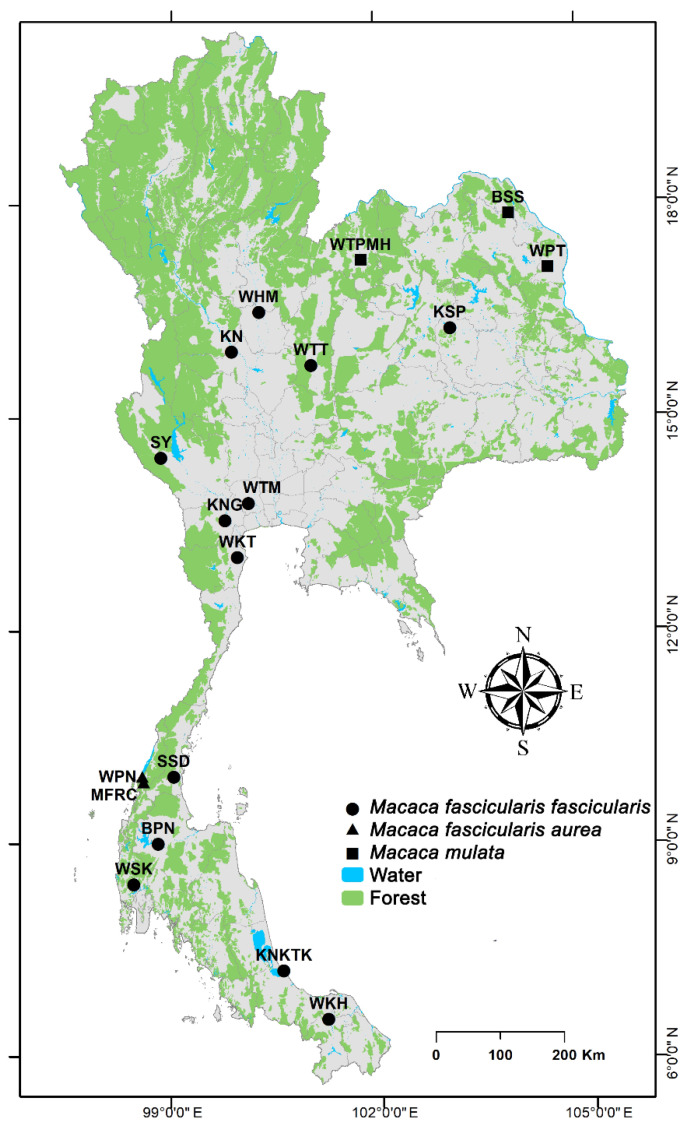
Geographical locations of 18 macaque populations in Thailand. BPN: Baan Pak Nam, Suratthani; BSS: Baan Sang School, Buengkarn; KN: Khao Nor, Nakhonsawan; KNG: Khao Ngu, Ratchaburi; KNKTK: Khao Noi Khao Tangkuan, Songkhla; KSP: Kosumpi, Mahasarakham; MFRC: Mangrove Forest Research Center, Ranong; SSD: Somdet Phra Sinagarindra Park, Chumphon; SY: Sai Yok, Kanchanaburi; WHM: Wat Haad Moon, Pichit; WKH: Wat Kuha Pimuk, Yala; WKT: Wat Khao Thamon, Petchaburi; WPN: Wat Pak Nam, Ranong; WPT: Wat Pattanajit, Nakhon Phanom; WSK: Wat Suwan Khuha, Pang-nga; WTM: Watthammasala, Nakhon Pathom; WTPMH: Wat Tham Pa Mak Ho, Loei; and WTT: Wat Tham Thep Ban Dan, Phetchabun.

Peripheral white blood cells (WBCs) of 377 wild individuals were obtained from 18 populations of long-tailed and rhesus macaques in Thailand. Detailed information on the sampled individuals is presented in Supplementary Table S1 and Figure 1. Morphospecies identification was performed as described in the previous studies [60,61,62,63]. Blood samples were collected from the femoral vein using a 22-gauge needle attached to 10 mL disposable syringes. These contained either 10 mM ethylenediaminetetraacetic acid for DNA extraction or 75 USP unit/mL heparin for cell culture. Permission for capturing and specimen collection in wild animals was granted by the Department of National Parks, Wildlife and Plant Conservation (DNP) of Thailand (approval no. TS.0909.704/2932, 2 July 2015). The experimental protocol for animal care and use was approved by the Institutional Animal Care and Use Committee of the Faculty of Science, Chulalongkorn University, Thailand (Protocol Review no. 1423010). After blood collection, all samples were centrifuged at 1000× *g* for 10 min, and the buffy coat containing white blood cells was harvested for DNA extraction. Total genomic DNA was extracted in accordance with the standard phenol–chloroform method [64]. DNA quality and concentration were determined using 1% agarose gel electrophoresis and a NanoDrop™ 2000 Spectrophotometer (Thermo Fisher Scientific, Wilmington, DE, USA), before being used as template for the construction of a DNA library to store pools of clones for molecular cloning by PCR.

### 2.2. Fosmid DNA Library and Isolation of Satellite DNA Sequences

A genomic library was constructed using a pCC1FOS fosmid library construction kit (Epicentre Biotechnologies, Madison, WI, USA), following the manufacturer’s protocol. Genomic DNA fragments of 35–45 kb were mechanically sheared and inserted into the 8.1 kb fosmid pCC1FOS vector. A total of 768 colonies from the long-tailed macaque genomic library were cultured in LB broth liquid medium (TM Media, Titan Biotech Ltd., Rajasthan, India) with 100 mg/mL Chloramphenicol solution (Sigma-Aldrich Co., St. Louis, MO, USA) and distributed into 96-well plates. Subsequently, 1 μL of each 100 µL culture was dotted onto nylon membranes. Genomic DNA from long-tailed macaques, which was mechanically sheared to an approximate size of 20 kb, was directly labeled using an AlkPhos direct labeling kit (GE Healthcare, Little Chalfont, UK), before being hybridized onto the membranes at 55 °C. Chemiluminescent signals were detected using the CDP-Star detection system (GE Healthcare) and captured on KODAK T-MAT G/RA dental film (Carestream Health, Rochester, NY, USA). Colonies with intense signals were selected, and those containing inserted DNA fragments of cen-satDNA sequences were screened using fluorescence in situ hybridization (FISH) mapping on chromosomes.

### 2.3. Cell Culture and Chromosome Preparation

Lymphocytes from 2 male long-tailed macaques were isolated from peripheral blood and cultured for 3 d in RPMI 1640 medium (Life Technologies, Grand Island, NY, USA) supplemented with 15% fetal bovine serum (FBS) (Life Technologies, NY, USA), 1% phytohemagglutinin (PHA15) (Remel, Lenexa, KS, USA), and 1% antibiotic-antimycotic solution (Life Technologies-Gibco, Carlsbad, CA, USA) as previously described, with slight modifications [65]. After 3 d, lymphocytes were subjected to treatment with 100 ng/mL colcemid (Life Technologies) for 45 min and fixed in 3:1 methanol/acetic acid (VWR International, LLC, Radnor, PA, USA) after hypotonic treatment in 0.075 M KCl (AppliChem GmbH, Ottoweg, Darmstadt, Germany) before being harvested. Cell suspensions were dropped onto clean glass slides and air-dried. Slides were kept at −80 °C until use.

### 2.4. C-Banding

C-banding was performed to examine the chromosomal distribution of constitutive heterochromatin using the standard barium hydroxide/saline/Giemsa method [66] with slight modifications. Briefly, chromosome slides were treated with 0.2 N HCl (Merck KGaA, Darmstadt, Germany) at 25 °C for 60 min and then with 5% Ba(OH)_2_ (Merck KGaA) at 50 °C for 1.30 s, followed by treatment with 2× SSC (Sigma-Aldrich Co.) at 65 °C for 60 min.

### 2.5. Fluorescence In Situ Hybridization Mapping of Cen-satDNA

The chromosomal locations of cen-satDNA were determined using FISH as previously described [67,68]. In particular, 250 ng of satDNA fragments were labeled by nick translation incorporating biotin-16-dUTP (Roche Diagnostics, Basel, Switzerland) according to the manufacturer’s protocol, and ethanol-precipitated with salmon sperm DNA and *Escherichia coli* tRNA. After hybridization of the biotin-labeled probes to long-tailed macaque chromosomes, the probes were detected by incubating the chromosome slides with fluorescein isothiocyanate-labeled avidin (avidin-FITC; Invitrogen, Carlsbad, CA, USA). Slides were subsequently stained with 1 µg/mL 4′, 6′-diamidino-2-phenylindole (DAPI) (Invitrogen). Fluorescence hybridization signals were captured using a cooled Charge-Coupled Device (CCD) camera mounted on a ZEISS Axioplan2 microscope (Carl Zeiss Microscopy GmbH, Kistlerhofstrasse, Munich, Germany) and processed using the MetaSystems ISIS v.5.2.8 software (MetaSystems, Altlussheim, Germany).

### 2.6. Centromeric Satellite DNA Sequencing in Macaques

Fosmid clones with intense signals were mapped on the centromeric region, and the nucleotide sequences of inserted DNA fragments at end-terminals were determined by the DNA sequencing service of First Base Laboratories Sdn Bhd (Seri Kembangan, Selangor, Malaysia) using universal M13 primers (M13F-pUC (−40): 5′-GTTTTCCCAGTCACGAC-3′ and M13R (−20): 5′-GCGGATAACAATTTCACACAGG-3′). The BLASTn program (http://blast.ncbi.nlm.nih.gov/Blast.cgi, accessed on 1 December 2021) was used to search nucleotide sequences in the National Center for Biotechnology Information (NCBI) database to confirm the identity of the amplified DNA fragments. Individual monomers were identified within multimers to design a specific primer pair to amplify cen-satDNA sequences in long-tailed and rhesus macaques. Nucleotide sequences were searched for homologies using the BLASTn program (http://blast.ncbi.nlm.nih.gov/Blast.cgi, accessed on 1 December 2021) and for patterns of repetitive sequences using RepBase (https://www.girinst.org/repbase/, accessed on 1 December 2021) [69]. Sequence alignment between a candidate clone-derived cen-satDNA from long-tailed macaque and a database-derived cen-satDNA repeat of rhesus macaque (accession number X04006) was performed to design specific primers. Amplification of cen-satDNA was carried out using the designed primers containing specific (Illumina) sequencing adaptors on their 5′ ends; Censat F: 5′-TCGTCGGCAGCGTCAGATGTGTATAAGAGACAGCTGTGAAATCATCTCACAGAG-3′; Censat R: 5′-GTCTCGTGGGCTCGGAGATGTGTATAAGAGACAGCTTTCTCTTTGTTATCGGAGG-3′. Each 15 μL PCR reaction contained 1× ThermoPol^®^ buffer, 1.5 mM MgCl_2_, 0.2 mM dNTPs, 0.2 μM of each primer, 0.5 U Taq polymerase (Apsalagen Co., Ltd., Bangkok, Thailand), and 25 ng genomic DNA. PCR conditions were as follows: initial denaturation at 94 °C for 4 min, followed by 35 cycles of 94 °C for 30 s, 63 °C for 45 s, 72 °C for 30 s, and a final extension at 72 °C for 10 min. PCR products were reamplified using the Nextera dual-index primer set (Supplementary Materials Table S1). Each primer contained a unique 6 bp index sequence. Each sample was amplified using a different combination index to allow multiplexing of up to 384 samples per run. The conditions of the second PCR reaction were as follows: initial denaturation at 94 °C for 4 min, followed by 35 cycles of 94 °C for 30 s, 55 °C for 45 s, 72 °C for 30 s, and a final extension at 72 °C for 10 min. PCR products were purified using the FavorPrep GEL/PCR purification mini kit (Favorgen Biotech Corp., Ping-Tung, Taiwan) and subsequently pooled at equal amounts based on the concentration measured by Quant-iT™ dsDNA HS assay kits (Invitrogen, Waltham, MA, USA). The pool of DNA libraries (size 340–350 bp) from 377 samples was paired-end sequenced (2 × 250 bp) on an Illumina MiSeq platform (Illumina, Inc., San Diego, CA, USA). The obtained nucleotide sequences were inputted in the BLASTn program (http://blast.ncbi.nlm.nih.gov/Blast.cgi, accessed on 1 December 2021) to search for nucleotide sequences in the National Center for Biotechnology Information database to confirm the identity/similarity of the amplified DNA fragments. The sequences generated were deposited in the Dryad repository (https://doi.org/10.5061/dryad.w3r2280sw, accessed on 19 May 2022).

### 2.7. Identification of Putative Satellite DNA in Long-Tailed and Rhesus Macaque Populations

To identify the occurrence of satDNA sequences in long-tailed and rhesus macaque populations, all Illumina reads were applied to a tandem repeat analyzer (TAREAN) [70], and sequence formats were generated for further analyses. All sequence data (Illumina reads) from the 377 samples were merged into a single FASTA file, which was used as input in the RepeatExplorer2 pipeline using default parameters that detected sequence similarities and variability by examination of reads, clusters, and graph topology [71]. RepeatExplorer is a computational pipeline for characterization of repetitive sequences and identification of centromeric repeats from unassembled next generation sequencing (NGS) data. RepeatExplorer also includes TAREAN tool for detecting putative satDNA sequences based on circular structures in their cluster graphs. Putative satDNA sequences were subsequenttly detected by the presence of circular structures in their cluster graphs. Consensus sequences of satDNA sequences were constructed from the most prevalent k-mers obtained by decomposing read sequences from the respective groups. The most frequent variants of sequences were reconstructed from the count of k-mers in the set of oriented reads [70]. Variants of satDNA monomers were selected based on multiple criteria: (i) by converting k-mer sequences constituting De Bruijin graphs, (ii) alignment of sequences with the same length, (iii) calculating consensus, and (iv) position probability matrix from k-mer weights. The output groups of the satDNA sequences were annotated using RepeatMasker to further confirm the occurrence of satDNA sequences [72]. All Illumina reads were assembled using Geneious Prime (version 2022.0.2) (http://www.geneious.com, accessed on 1 December 2021) [73] and individual contigs were generated using a custom sensitivity method with default parameters [73]. Sequence features including GC content, sequence length, pairwise identity, and consensus length were determined for each assembled contig using Geneious Prime. To elucidate the relationship of the 2 datasets derived from k-mer and assembled contig approaches, multiple sequence alignment of putative satDNA sequences was performed using multiple sequence comparison by log-expectation (MUSCLE version 3.8.31) (http://www.ebi.ac.uk/Tools/msa/muscle/, accessed on 1 December 2021) [74] with default parameters. Sequences were clustered using maximum composite likelihood analysis with neighbor-joining phylogeny and a total of 1000 bootstrap replications following the default parameters of MEGA11: Molecular Evolutionary Genetics Analysis (version 11.0.10) [75]. Evolutionary distances were computed using the number of base substitutions per site, and all ambiguous positions were removed for each sequence pair (pairwise deletion option). The phylogenetic trees were then annotated and edited using Interactive Tree of Life (iTOL) version 4 [76]. Tajima’s *D* neutrality test (D) [77] was performed using MEGA11 with a substitution model type of “nucleotide” to examine whether cen-satDNA sequences had evolved randomly (“neutrally”).

### 2.8. Genomic Organization and Comparative Genomics of Satellite DNA Sequences

BLASTn of satDNA sequences was performed to screen their corresponding genomic regions across the 2 macaque genomes. The chromosomal level genome assemblies of long-tailed and rhesus macaques (NCBI accessions: GCA_012559485.3 and GCA_003339765.3, respectively) were retrieved from the NCBI assembly database and unplaced scaffolds were eliminated to restrict the analysis for chromosomes. Random hits with alignment size less than 200 bp and percent identity less than 80% were also filtered out. The repeat density of mapped cen-satDNA was calculated and the number of intervals (chromosomal blocks/loci of a specific size) for the region spanning 1 megabase pair (Mbp) was counted using BEDTools version 2.29.2 [78]. Chromosomes with the highest repeat density in the respective regions were identified and selected to investigate the complex structures of satDNA arrangements in these regions. The StainedGlass pipeline workflow [79] was applied and implemented in Snakemake [80,81]. All possible pairwise alignments between satDNA sequences and respective chromosomal regions were computed using Minimap2 [82], with outputs being generated as heatmap plots. All satDNA sequences were also compared with the human genome (GCA_000001405.28) using the BLAT tool [83], with default parameters in Ensembl to identify the patterns of sequence distribution on chromosomes, resulting in the generation of an ideogram. The default parameters in Ensembl were set as “alignment BLASTn, Search sensitivity normal, Maximum number of hits to report 100, and Match/Mismatch scores 1,-3”. The human genome was the only available genome with complete karyotypic information necessary for ideogram construction. 

The assembled satDNA contigs of long-tailed and rhesus macaques were also searched in Ensembl genome browser version 105 (https://www.ensembl.org/, accessed on 1 December 2021) [84] for putative orthologs across primate genomes. The mapping hits of all randomly searched consensus sequences had the same distribution across genomic regions in primates. Therefore, we selected a single consensus assembled sequence of satDNA from both macaque species as representative sequences for comparative analysis. The satDNA sequences were aligned against genomes of selected available primate species in Ensembl using BLAST. The default parameters and sequence length variability of orthologous sequences were observed from the alignment output file. This file was generated as a tabular format from the BLASTn hits, with records of percent identity and alignments size of the query satDNA sequence for each mapped region in primate genomes. Random hits were filtered out and the distributions of homologous (mapped) regions with significant hits (percent identity > 95% and E-value < 1e^−50^) were plotted as boxplots using “ggplot2” [85] in R [86] to show the variability in sequence length. A total of 17 primate species were considered, including northern white-cheeked gibbon, black-capped squirrel monkey (*Saimiri boliviensis*, Geoffroy and Blainville, 1834) [87], human (*Homo sapiens*, Linnaeus, 1766) [88], Bornean orangutan (*Pongo pygmaeus*, Linnaeus, 1766) [88], chimpanzee [35], bonobo (*Pan paniscus*, Schwarz, 1929) [89], western gorilla (*Gorilla gorilla*, Savage, 1847) [36], Ugandan red Colobus (*Piliocolobus tephrosceles*, Elliot, 1907) [90], black snub-nosed monkey (*Rhinopithecus bieti*, Milne-Edwards, 1897) [91], vervet monkey (*Chlorocebus sabaeus*, Linnaeus, 1766) [88], Angola colobus (*Colobus angolensis*, Sclater, 1860) [92], drill (*Mandrillus leucophaeus*, Cuvier, 1807) [93], gelada (*Theropithecus gelada* (Rüppell, 1835), olive baboon (*Papio Anubis*, Lesson, 1827), rhesus macaque, long-tailed macaque, and southern pig-tailed macaque (*Macaca nemestrina*, Linnaeus, 1766) [88].

### 2.9. Tests of Genetic Diversity within and between Macaque Populations

To perform genetic diversity analyses within/between the 18 macaque populations, BLAST of the consensus sequences of satDNA sequences was performed against the “nr” database in NCBI and the best hit selected as the reference sequence was retrieved. Sequence reads of all macaques were aligned to the reference sequence using the Burrows–Wheeler Aligner (version 0.7.10-r789) [94] and the Sequence Alignment/Map (SAM) format files in Binary Alignment Map (BAM) and were converted using SAMtools (version 0.1.19) [95], followed by sorting and importing to the STACKS software pipeline (version 2.60) [96] for variant calling used to build variant loci and generate SNP loci. The SNP loci from BAM files were subsequently transformed to variant calling format (VCF) files, which were then used for downstream analyses of genetic diversity within and between macaque populations. Different file formats for data analyses were generated using PGDSpider (version 2.1.1.5) [97] and dartR package (version 1.9.9.1) [98]. The genetic diversity of macaque populations was evaluated using different parameters, including the number of different alleles (*N_a_*), number of effective alleles (*N_e_*), Shannon’s information index (*I*), expected heterozygosity (*H_e_*), observed heterozygosity (*H_o_*), unbiased expected heterozygosity (*uHe*), and fixation index (*F*), which were calculated using GenAlEx (version 6.5) [99]. To investigate similarities between individuals and the state of populations, pairwise *F*_ST_ genetic distances between the 18 populations were calculated using GenAlEx (version 6.5) [99]. A value of *F_ST_* = 0.25 indicated large differentiation between populations, a value in the 0.15–0.25 range indicated high differentiation, and a value in the 0.05–0.15 range indicated moderate differentiation, whereas it was negligible if *F_ST_* was 0.05 or less [100]. To facilitate understanding of the genetic differences between the 18 populations, hierarchical analyses of molecular variance (AMOVA) [101] were performed using GenAlEx (version 6.5) [99]. This provided hierarchical partitioning of the total genetic variation within individuals, between individuals, between populations, and the estimation of *F*-statistics, which were tested using random permutation. To test the homogeneity of the population examined, principal coordinates analysis (PCoA) was conducted in GenAlEx (version 6.5) and data were visualized using “ggplot2” [85] in R [86]. Bayesian clustering analysis was also performed to infer the number of genetic clusters for all samples. We ran STRUCTURE from *K* = 1 to *K* = 10 with 10 iterations per *K*, a 10,000 generation burn in, and 50,000 Markov Chain Monte Carlo steps per iteration. STRUCTURE runs were implemented without previous information about group membership. An estimate of the optimal number of genetic clusters (*K*) was obtained using the method by Evanno et al. (2005) [102] implemented in STRUCTURE HARVESTER (version 0.6.94) [103]; meanwhile, results were compared across various *K* values regardless of the optimal *K* to assess the substructuring present within the data. Demographic history was determined using the statistical test of neutrality [77,104]. Tajima’s *D* [77] and Fu and Li’s D* and F* tests [104] were calculated using PopGenome (version 2.7.5) in R package [105], while the jackknife resampling function, which is part of this package, was applied for empirical *p*-value determination [105,106]. The genetic structure of each dataset was examined in relation to its geographic location using a Mantel test [107], implemented in Alleles In Space (AIS) [108], with 1000 permutations to establish the significance of the correlation coefficient (null hypothesis: genetic clustering as a result of isolation-by-distance, *p* < 0.01). To visualize spatial patterns of genetic diversity, we conducted a landscape shape interpolation (LSI) analysis, wherein geographic coordinates (*x*- and *y*-axes) can be related to genetic distances (surface plot heights, *z*-axis) in a three-dimensional surface plot [108]. Peaks in the surface plot represented areas of high genetic distance between individuals and were considered areas of restricted genetic exchange. Troughs in the surface plot revealed areas of low genetic distance. We performed the LSI analysis in the AIS software, using a distance weighting parameter (α) of 1 and grid settings of 80 × 80 grid were specified. All analyses implemented in AIS used sequences as the input matrix (raw genetic distances) and Universal Transverse Mercator coordinates ArcGis were also used to construct maps for each population dataset and to incorporate information on cluster membership [109].

## 3. Results

### 3.1. Karyotype and C-Positive Heterochromatin of Long-Tailed Macaque

**Figure 2:**
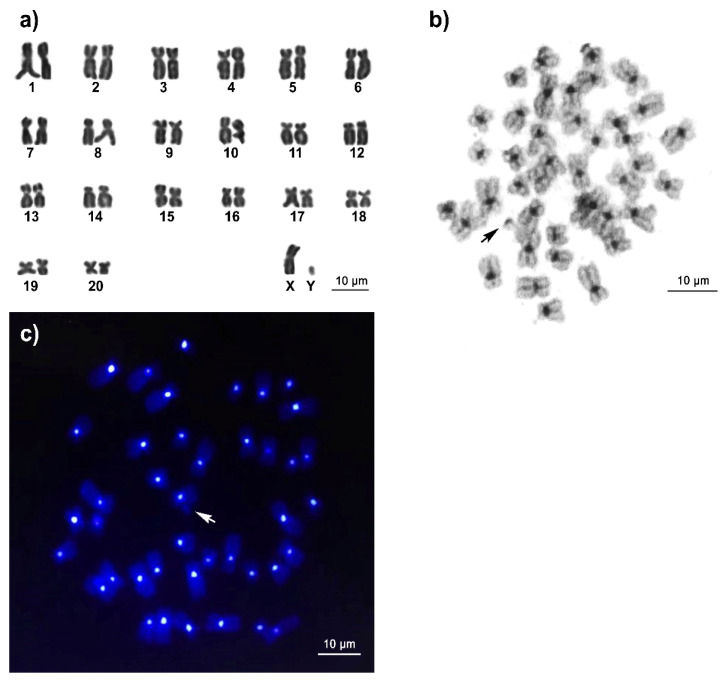
Karyotype and chromosomal locations of cen-satDNA in the genome of male *Macaca fascicularis*, Raffles, 1821. Giemsa (**a**), C-banded metaphase spread (**b**), and FISH patterns of the cen-satDNA clone on DAPI-stained metaphase chromosome spreads in the genome of male *Macaca fascicularis*, Raffles, 1821 (**c**). Arrows indicate Y chromosome. Scale bars represent 10 μm.

We performed Giemsa-stained metaphase spreads of long-tailed macaque cells, which revealed a chromosome number of 2n = 42 (FN = 83 in males and 84 in females), comprising 7 pairs of large metacentric (1st, 13th, 15th, 16th, and 18–20th) and 13 pairs of submetacentric (2nd–12th, 14th, and 17th) chromosomes. The male karyotype contained the metacentric X chromosome and a small metacentric Y chromosome (Figure 2a). In addition, we detected the presence of C-positive heterochromatin in the centromeric region of all chromosomes (Figure 2b).

### 3.2. Isolation of Highly Repetitive DNA Sequences and Their Nucleotide Sequences

**Figure 3:**
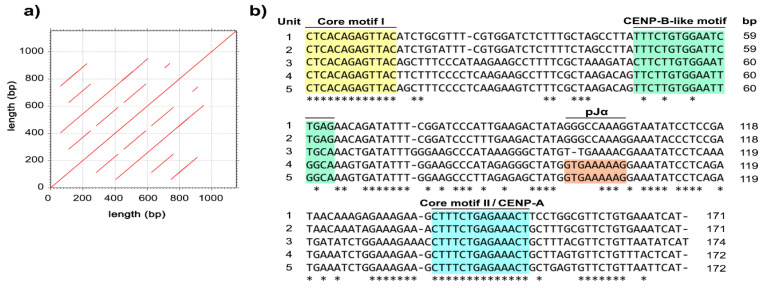
Dot plot comparison of the fosmid clone sequence against itself (**a**). Alignment of 5 cen-satDNA monomers from fosmid clones with intense signals mapped on the centromeric region (**b**).

We then performed genomic hybridization using 768 fosmid clones and identified 14 prominent clones potentially containing highly repetitive sequences. To investigate their chromosomal localization, we mapped all prominent clones to long-tailed macaque chromosomes. We observed more than 20 metaphase spreads, with hybridization efficiencies ranging from 70% to 90%. We detected that all the prominent clones were localized to the centromeric regions of all the chromosomes, except for the Y chromosome (Figure 2c). We then characterized the 500–800 bp nucleotide sequences from the terminal ends of 2 out of 14 randomly prominent clones using the M13 universal primers. Dot matrix analysis showed that these two clone sequences contained tandem-arrayed repetitive sequences (Figure 3a). Within the clone insert, we detected five units with lengths ranging from 170 to 174 bp, and GC contents ranging from 36.8% to 41.5%, with an average of 39.5%, which were characterized by a secondary structure (Supplementary Figure S1). We therefore designated these satDNA as cen-satDNA sequences. We identified the following conserved motifs in all the units in the cen-satDNA sequences: CTCACAGAGTTAC termed “core motif1, CM1” and CTTTCTGAGAAACT termed “core motif2, CM2” (Table 1 and Figure 3b). We searched the NCBI database for sequence homology to any of the families of repetitive sequences and found that our detected cen-satDNA sequences showed sequence similarity to alpha satDNA in human, rhesus macaque, bonnet macaque (*Macaca radiata*) (É. Geoffroy, 1812) [110,111], and olive baboon (Lesson, 1827) [37]. We further observed that these alpha-satDNA sequences showed high sequence similarity with the nucleotide sequences of conserved centromeric motifs in the human genome, such as the pJα motif (100% identity), centromere-binding protein A (CENP-A) box (64.29% identity) located in the CM2, and CENP-B box (37.50–87.50% identity) [112] (Figure 2). We also performed a Repbase [69] search using the cen-satDNA sequences and identified a partial sequence with 77.39% identity to the ALRa-SAT satellitee from primates (122 bp, GC content: 41%) that was also found in human, rhesus macaque, Sumatran orangutan, chimpanzee, and northern white-cheeked gibbon (*Nomascus leucogenys*, Ogilby, 1840) [113].

### 3.3. Striking Sequence Variability of Cen-satDNA in Macaque Populations

**Figure 4:**
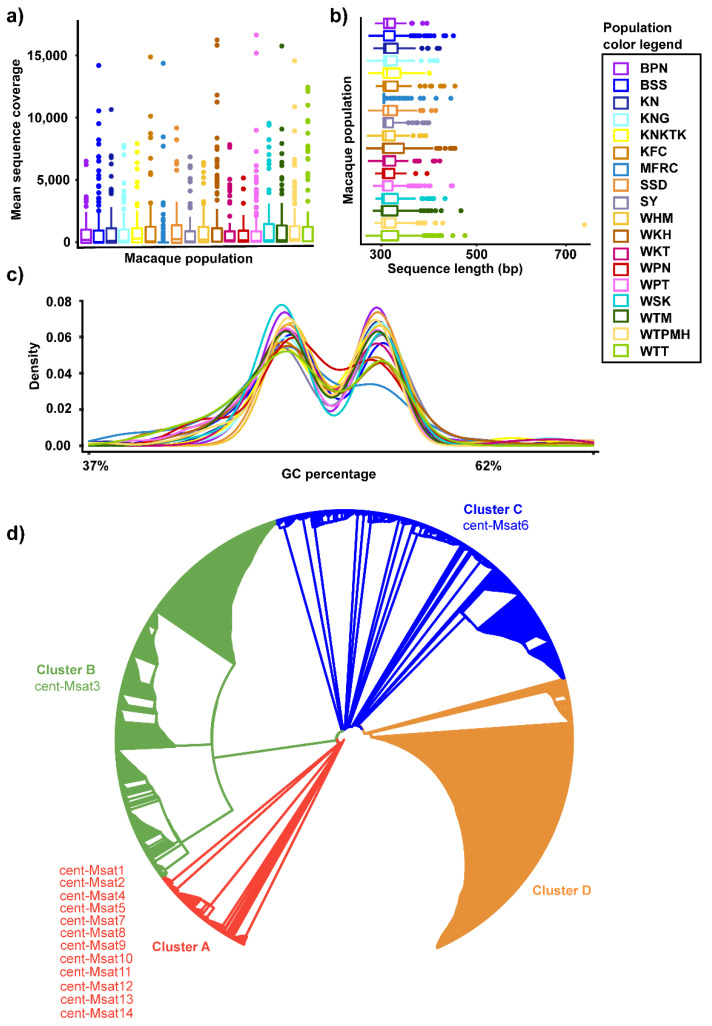
Characterization of variable features and coevolved clusters of cen-satDNA in the genome of macaque populations. Read depth and sequence length variations of cen-satDNA in macaques at a population scale. Distribution of sequence reads mean coverage (**a**) and sequences length across different populations (**b**). Density plot of GC% indicates a diverse range of GC composition among sequences, with the bar chart displaying the population-wise relative abundance of consensus sequences of cen-satDNA with different sizes (**c**). An unrooted phylogenetic tree using 1546 consensus sequences derived from the assembly of sequences from all 377 macaque samples revealed 4 distinct clusters. Dense branching patterns within each cluster are indicative of concerted evolution in cen-satDNA heterochromatic arrays. Evolutionary history was inferred using the Neighbor-Joining method. Tree is drawn to scale, with branch lengths in the same units as those of the evolutionary distances used to infer the phylogenetic tree (**d**). BPN: Baan Pak Nam, Suratthani; BSS: Baan Sang School, Buengkarn; KN: Khao Nor, Nakhonsawan; KNG: Khao Ngu, Ratchaburi; KNKTK: Khao Noi Khao Tangkuan, Songkhla; KSP: Kosumpi, Mahasarakham; MFRC: Mangrove Forest Research Center, Ranong; SSD: Somdet Phra Sinagarindra Park, Chumphon; SY: Sai Yok, Kanchanaburi; WHM: Wat Haad Moon, Pichit; WKH: Wat Kuha Pimuk, Yala; WKT: Wat Khao Thamon, Petchaburi; WPN: Wat Pak Nam, Ranong; WPT: Wat Pattanajit, Nakhon Phanom; WSK: Wat Suwan Khuha, Pang-nga; WTM: Watthammasala, Nakhon Pathom; WTPMH: Wat Tham Pa Mak Ho, Loei; and WTT: Wat Tham Thep Ban Dan, Phetchabun.

We grouped all the available reads of each population (total 1,764,463 sequences from 377 individuals) using RepeatExplorer and TAREAN. We then assessed the mean read coverage for all 18 populations and recorded deep coverage sufficient to obtain an accurate contig assembly and population-scale sequencing for all individuals across the populations, with a mean coverage exceeding 100× for most individuals (Figure 4a). Using TAREAN, we detected five different k-mer with the most frequent being 27-mer repeats, including 265,060 monomers obtained by decomposing the read sequences from the corresponding groups. We observed 14 distinct cen-satDNA subfamilies with variable monomers (Supplementary Figure S2 and Supplementary Table S3). We noticed that the cent-Msat9 and cent-Msat3 subfamilies showed the highest and lowest abundance (2003 and 501 frequency) across the assembled contigs. We also detected cen-satDNA with a 96.02% level of abundance using RepeatMasker, further validating the prediction of the cen-satDNA subfamilies detected by TAREAN [114]. To confirm the occurrence of sequence variability derived from the NGS reads with highly complex satDNA, we analyzed the same datasets obtained from sequencing using contig assembly. We thus obtained new sequences of monomer units that were highly similar to the alpha-satDNA sequences isolated from the genomic library (more than 88%). We observed 1546 contigs of the cen-satDNA sequences, with remarkable variable lengths ranging from 293 to 718 bp (average size 318 bp) across all individuals (Supplementary Dataset S1).

We specifically found that the rhesus macaque population of Wat Tham Pa Mak Ho, Loei (WTPMH), showed the highest variability of cen-satDNA sequences, whereas the long-tailed macaque population of Sai Yok, Kanchanaburi (SY), had the lowest range of variation in cen-satDNA sequence size (Figure 4b). Remarkably, we noticed that the GC content of these cen-satDNA sequences was highly variable, ranging from 37% to 63% across all macaque populations (Figure 4c). Tajima’s neutrality test recorded a D value of −1.59391, measured across all populations, which was not statistically significant, indicating a possible recent selective sweep of cen-satDNA in macaques (Table 2). We subsequently reconstructed an unrooted phylogenetic tree to infer the evolutionary relationships and identify the putative clusters using all the cen-satDNA sequences. We specifically defined these clusters according to a set of particular nucleotide substitutions. We grouped all the sequences together into four major clusters: A, B, C, and D. (Figure 4d). Finally, we examined these 14 subfamilies for their relationship with the four major clusters. We detected that 10 out of 14 subfamilies corresponded to cluster A, whereas cluster B and cluster C contained a single subfamily, cen-Msat3 and cen-Msat6, respectively (Figure 4d). We did not observe any cen-satDNA subfamilies derived from the k-mer analysis in cluster D.

### 3.4. Genomic Organization of Cen-satDNA Sequences

**Figure 5:**
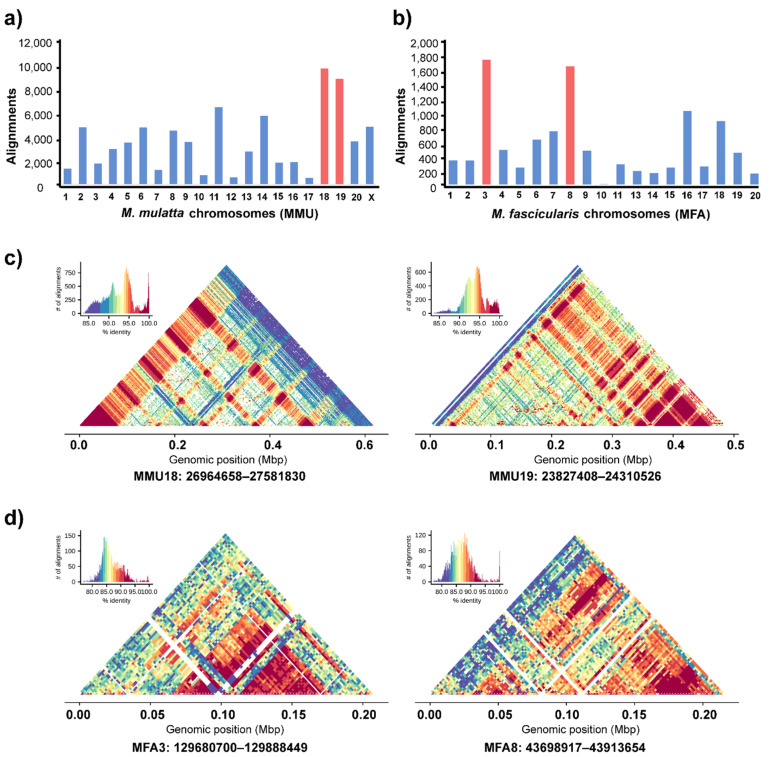
Evolutionary and structural landscape of cen-satDNA within macaque genomes and centromeric complexes. Number of significant alignments per chromosomes of *M. Mulatta* (**a**) and *M. Fascicularis* (**b**). Chromosomes with the 2 most abundant hits are highlighted in red and were selected for further investigation of structural organization. Centromeric regions identified using StainedGlass are visualized as heatmap plots for representative regions in the genome of *M. mulatta* (chromosomes 18 and 19) (**c**) and *M. fascicularis* (chromosomes 3 and 8) with different patterns of cen-satDNA arrays (**d**).

We also examined the organization of cen-satDNA sequences in the centromeric regions of long-tailed and rhesus macaque chromosomes using comparative genomic analysis. Using in silico mapping, the cen-satDNA sequences with all subfamilies and clusters were mapped onto all chromosomes, except for the Y chromosome (with no alignments). We found that the total number of “filtered nonrandom alignments” (percent identity > 80 and alignment length > 200 bp) was higher in rhesus macaques (total 74,478) compared with that in long-tailed macaques (total 10,272) (Figure 5a,b). Moreover, we observed a repetitive density and enriched regions of cen-satDNA with the highest abundance on chromosomes 3 and 8 of long-tailed macaques, with 1726 and 8724 total repeats, respectively, and on chromosomes 18 and 19 of rhesus macaque, with 9575 and 8724 total repeats, respectively (Supplementary Table S3). We then visualized the complex tandem arrangements of cen-satDNA sequences in the enriched centromeric regions of these four chromosomes using a heatmap (Figure 5c,d). Accordingly, we identified different patterns of arrangement of the cen-satDNA sequences in each centromeric region of different chromosomes in both species, indicating their high structural complexity on the chromosomes (Figure 5c,d, Figures S3 and S4).

**Figure 6:**
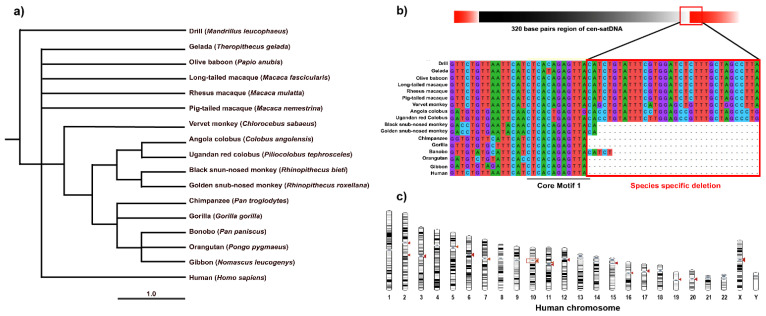
Evolutionary relationship of cen-satDNA in different primates. (**a**) Neighbor-Joining method-based phylogeny of cen-satDNA across primate species. (**b**) Visualization of multiple sequence alignment of derived orthologs for cen-satDNA from analyzed species showing specific deletions and interspecific divergence in apes. (**c**) Chromosome localization of cen-satDNA on human chromosome revealed by Genomicus v100.01 [117].

We compared the cen-satDNA consensus sequences from the assembled contigs with orthologous sequences from 17 primate species using multiple sequence alignment to provide insights into the polymorphically and evolutionarily diverged sites. We observed a species-specific 120 bp deletion downstream of CM1 (Figure 6b). In particular, we detected that the vervet monkeys (*Chlorocebus sabaeus*, Linnaeus, 1766) [88], geladas, olive baboons, rhesus macaques, long-tailed macaques, and southern pig-tailed macaques showed long cen-satDNA sequences, whereas the western gorilla or Sumatran orangutan contained shorter cen-satDNA sequences (Supplementary Figure S5). By contrast, we did not observe any similarity between cen-satDNA sequences in certain primate genomes, including that of the common marmoset (*Callithrix jacchus*, Linnaeus, 1758) [115], bamboo lemur (*Prolemur simus* Gray, 1871) [116], and tarsier (*Carlito syrichta*, Linnaeus, 1758) [115] (Supplementary Dataset S2). For comparison with the human genome, we mapped these cen-satDNA sequences on autosomes and the X chromosome but not on the Y chromosome (Figure 6c).

### 3.5. Genetic Diversity within and between Macaque Populations

**Figure 7:**
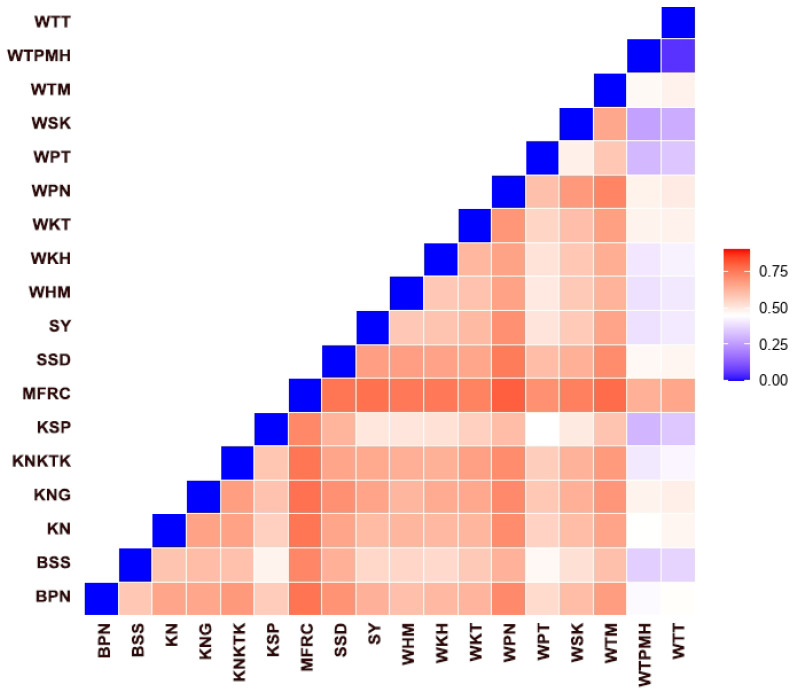
Pairwise *F*_st_ in the genome of 18 macaque populations. BPN: Baan Pak Nam, Suratthani; BSS: Baan Sang School, Buengkarn; KN: Khao Nor, Nakhonsawan; KNG: Khao Ngu, Ratchaburi; KNKTK: Khao Noi Khao Tangkuan, Songkhla; KSP: Kosumpi, Mahasarakham; MFRC: Mangrove Forest Research Center, Ranong; SSD: Somdet Phra Sinagarindra Park, Chumphon; SY: Sai Yok, Kanchanaburi; WHM: Wat Haad Moon, Pichit; WKH: Wat Kuha Pimuk, Yala; WKT: Wat Khao Thamon, Petchaburi; WPN: Wat Pak Nam, Ranong; WPT: Wat Pattanajit, Nakhon Phanom; WSK: Wat Suwan Khuha, Pang-nga; WTM: Watthammasala, Nakhon Pathom; WTPMH: Wat Tham Pa Mak Ho, Loei; and WTT: Wat Tham Thep Ban Dan, Phetchabun.

**Figure 8:**
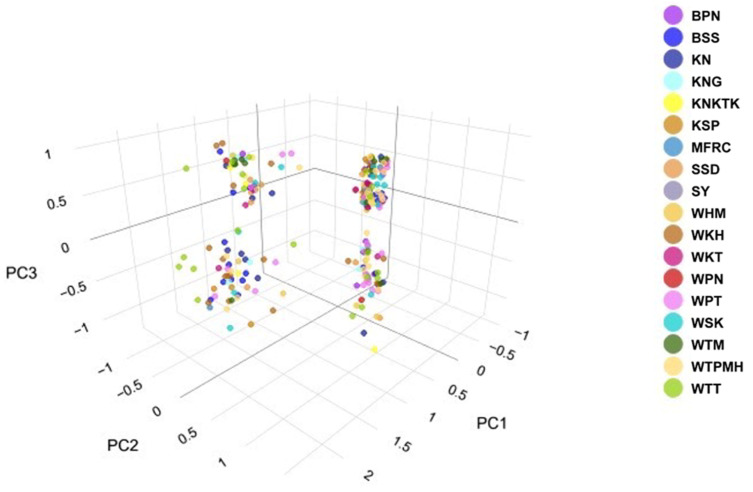
Scatter plot of PCoA from 377 individuals colored according to populations. BPN: Baan Pak Nam, Suratthani; BSS: Baan Sang School, Buengkarn; KN: Khao Nor, Nakhonsawan; KNG: Khao Ngu, Ratchaburi; KNKTK: Khao Noi Khao Tangkuan, Songkhla; KSP: Kosumpi, Mahasarakham; MFRC: Mangrove Forest Research Center, Ranong; SSD: Somdet Phra Sinagarindra Park, Chumphon; SY: Sai Yok, Kanchanaburi; WHM: Wat Haad Moon, Pichit; WKH: Wat Kuha Pimuk, Yala; WKT: Wat Khao Thamon, Petchaburi; WPN: Wat Pak Nam, Ranong; WPT: Wat Pattanajit, Nakhon Phanom; WSK: Wat Suwan Khuha, Pang-nga; WTM: Watthammasala, Nakhon Pathom; WTPMH: Wat Tham Pa Mak Ho, Loei; and WTT: Wat Tham Thep Ban Dan, Phetchabun.

**Figure 9:**
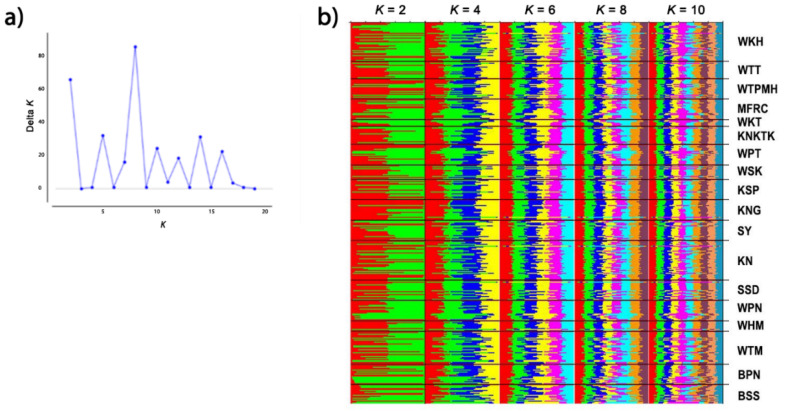
Population structure of 377 macaque individuals in Thailand. (**a**) Mean Ln P (*K*) graph. (**b**) Structure bar plots depicting model-based clustering results for inferred *K* = 2, 4, 6, 8, and 10. Each vertical bar on the *x*-axis represents an individual, while the *y*-axis represents the membership proportions (posterior probability) in each genetic cluster. BPN: Baan Pak Nam, Suratthani; BSS: Baan Sang School, Buengkarn; KN: Khao Nor, Nakhonsawan; KNG: Khao Ngu, Ratchaburi; KNKTK: Khao Noi Khao Tangkuan, Songkhla; KSP: Kosumpi, Mahasarakham; MFRC: Mangrove Forest Research Center, Ranong; SSD: Somdet Phra Sinagarindra Park, Chumphon; SY: Sai Yok, Kanchanaburi; WHM: Wat Haad Moon, Pichit; WKH: Wat Kuha Pimuk, Yala; WKT: Wat Khao Thamon, Petchaburi; WPN: Wat Pak Nam, Ranong; WPT: Wat Pattanajit, Nakhon Phanom; WSK: Wat Suwan Khuha, Pang-nga; WTM: Watthammasala, Nakhon Pathom; WTPMH: Wat Tham Pa Mak Ho, Loei; and WTT: Wat Tham Thep Ban Dan, Phetchabun.

**Figure 10:**
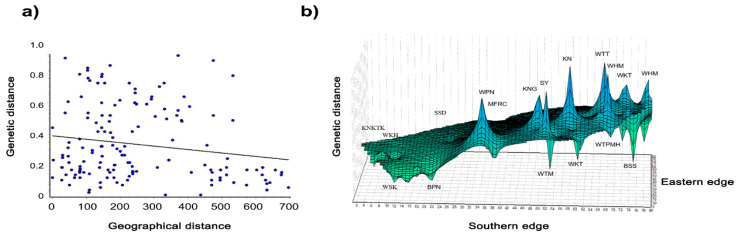
Correlation of genetic (mantel test) (**a**) and Genetic landscape interpolation plots (**b**) of 377 macaque individuals from 18 populations in Thailand depicting areas with high or low genetic differentiation based on geographic coordinates.

We recorded an alignment rate of 99.2% for the genomic sequences of 377 macaques using reference satDNA sequences and built 29 loci with a mean size of the genotyped sites of 301.34 bp and 520.6X as read coverage per locus. The dataset, with stacks for all populations, contained 1505 variants. We also inferred a phylogenetic tree of macaques from the single nucleotide polymorphisms (SNPs) of the cen-satDNA (Supplementary Figure S6). Consequently, we detected that the population exhibited *F* values of −0.510, while the *I* ranged from 0.110 to 0.359. The *H*_o_ values ranged from 0.133 to 0.382 (mean ± standard error [SE]: 0.193 ± 0.007), while the *H*_e_ values ranged from 0.078 to 0.241 (mean ± SE: 0.119 ± 0.004) (Table 3). Welch’s *t*-test showed that *H*_o_ was significantly different from *H*_e_ in all populations (Supplementary Dataset S3). A pairwise comparison of *H_o_* between the 18 populations revealed that 77% of the population pairs did not differ. This was also observed with a pairwise comparison of *H_e_* (Supplementary Dataset S3). The standard genetic diversity indices are summarized in Table 3. The pairwise *F*_ST_ values, which represent population structure based on the differences between the allele frequencies of the two populations, were represented as a heatmap plot (Figure 7). In case of *F*_ST_ > 0.25, a state of different populations was indicated. Our AMOVA analysis demonstrated 67% molecular variation (*p* < 0.001) within a population and 4% difference (*p* < 0.001) between populations (Table 4). In addition, PCoA revealed that the first, second, and third principal components accounted for 27.24%, 10.04%, and 7.69% of the total variation, respectively, and provided support for four tentatively differentiated groups (A, B, C, and D) as mentioned above (Figure 8). A Bayesian structural analysis revealed the highest posterior probability based on Evanno’s Δ*K*, with all macaque populations showing the genetic admixture of cen-satDNA sequences with all four major clusters (Figure 9). We used three neutrality tests (Tajima’s *D* values, Fu and Li’s F* values, and Fu and Li’s D* values) to observe the historical population expansion based on the cen-satDNA sequences. However, we noticed that not all values were statistically significant (Table 2). The Mantel test revealed no correlation between nucleotide diversities and geographic distance from isolation-by-distance (Figure 10a). Of note, our LSI analyses revealed high nucleotide diversities. In particular, the Khao Nor (KN) population belonging to *M*. *fascicularis* and the Wat Tham Thep Bandan (WTT) population belonging to *M*. *fascicularis* showed high nucleotide diversities (Figure 10b).

## 4. Discussion

Despite the importance of the centromere region, knowledge of the centromeric sequences remains limited, mainly because of the highly tandem repetitive and complex nature of these sequences. This obscures the process of genome sequencing and assembly. Although the potential impact of the variations in the centromeric sequences and their associated satellites on the functions of centromeres and heterochromatin has been recognized, their study remains difficult [118]. However, the recent integration of bioinformatics and cytogenetics as chromosomics has greatly assisted the assessment of repeat variations [119,120]. These combined approaches offer deep insights into the organization of cen-satDNA within primate genomes, thus facilitating our understanding of their possible roles in adaptive evolution at the population level and highlighting the extraordinary diversification of tandem repeats.

### 4.1. Turnover of Cen-satDNA Sequences with Multiple Subfamilies in Long-Tailed and Rhesus Macaque Populations

In this study, we found that the cen-satDNA sequences isolated from long-tailed and rhesus macaque centromeres were homologous to the alpha-satDNA family in primates. More specifically, they showed a tendency toward AT-rich monomers, characterized by secondary structures, including helices and stem-loops [39,121]. The limitations derived from the PCR-based approach drastically reduced the number of available sequences [122]. Genomic and phylogenetic characterizations provided information on the structural organization of each subfamily in each cluster. The co-occurrence of clusters and subfamilies in macaque populations and their interspecific divergence suggested that cen-satDNA followed the library model of satDNA evolution, in which different satDNA subfamilies coexist in the same genome due to amplification–contraction events of these sequence pools. However, this was not the case in the cen-satDNA observed in this study, where 14 satDNA subfamilies were retained within the both long-tailed and rhesus macaques. This rejected our hypothesis (i) as mentioned above, and the organization and diversity of cen-satDNA shared between the two macaque species indicated nonindependent evolution. The cen-satDNA remained in the common ancestor of the two species until at least 5.5 MYA [123]. The occurrence of large numbers of subfamilies in both long-tailed and rhesus macaques has probably been caused by the frequent turnover of the tandem repeat sequences [40]. This redundancy and high rate of change suggest that long-tailed and rhesus macaques contain substitutions, even in functional domains, without deleterious effects. The pattern of differentiation was also variable among repeats, revealing that some k-mers evolved relatively independently of others. Different cen-satDNA subfamilies underwent concerted evolution in abundance and might have retained essential protein-binding sites, structural domains, and sites for epigenetic modifications [124]. Cluster A, with many cen-satDNA subfamilies, adopted a monomeric organization, further supporting the hypothesis that centromeres are composed of repeated arrays evolved from simple monomeric structures [125,126]. Clusters B and C associated into multimers and possible parts of higher order regions (HOR), suggesting the differential evolution of tandem repetitive arrays in the genomes of long-tailed and rhesus macaques. Clusters B and C’s multimers are probably tandemly repeated or represent only a part of a longer HOR involving other monomers, as observed in other primates [28,29]. Higher-order structures might have a strict requirement for sequence length and the conservation of particular repeat regions [28,29,127,128,129]. Cluster D contained 316 bp of cen-satDNA and could not be classified in any subfamily, suggesting that it is an intermediate structural unit. This might have resulted from an ancient dimer of monomers and the subsequent deletion, substitution, and homogenization between the two monomers, followed by homogenization between monomers of nonhomologous chromosomes and the fixation of specific complex structures. An alternative explanation is that a monomer in the cluster D became amplified together with parts of anonymous adjacent monomer variants, and these spread together as a new repeated unit, not necessarily a HOR. The added part might be a favorable consequence of a monomer sequence from a degenerated short segment. For instance, an increase in repeated unit length and complexity through the merging of shorter repeat motifs or degenerated monomers has been found in satellite III on human chromosome 14 [130], human beta satellite DNA [131], and house mouse (*Mus musculus*, Linnaeus, 1758) [115,132,133].

Comparison of all cen-satDNA sequences derived from 1,764,463 sequences from 18 macaque populations revealed the existence of two conserved core sequence motifs, CM1 and CM2, which are also observed in alpha-satellite arrays in other primates [134,135]. This suggests that the two CMs are likely to be essential for the maintenance of chromosomes in primates, and can be expressed as *s* = 1, where *s* is the selection coefficient used in population genetics [136,137]. Comparing these cen-satDNA sequences with those of other primates revealed that similar cen-satDNA sequences were present in Cercopithecoidea (Old World monkeys) and Hominoidea (apes and human) but not in Ceboidea (New World monkeys) [138]. However, comparative genomics identified a 120 bp deletion downstream of CM1 in apes. In addition, CM2 was shown to be homologous to the CENP-A box, which is found at active centromeres [136,137]. The CENP-A box has rapidly evolved in many animals and CENP-A is a histone H3-like protein that binds to this box [139]. This suggests that the CENP-A box imposes a complementary selection pressure on cen-satDNA sequences to ensure protein–DNA compatibility [140,141]. By contrast, the pJα binding site was found to be retained in the repeated motif, with more than 90% of the number of cen-satDNA sequences from all populations, suggesting that it is nonessential with *s* ≠ 1, but highly positive for a higher chromosome stability of both long-tailed and rhesus macaques. Sequence regions similar to the pJα binding site have been found in some mammalian species as well as primates [142]. Similarly, the core sequences of the CENP-B box, an evolutionary conserved domain (ECD), were partially observed in most cen-satDNA sequences in this study, indicating the presence of CENP-B box-like boxes in long-tailed and rhesus macaques. Although not all ECD nucleotides are present in the CENP-B box-like motif found in the cen-satDNA sequence, we cannot exclude its functional activity. Such divergent motif sequences have also been observed in the cen-satDNAs of the African bush elephant (*Loxodonta africana*, Blumenbach, 1797) [143], the nine-banded armadillo (*Dasypus novemcinctus*, Linnaeus, 1758) [115,144], and the two-toed sloth of the *Choloepus* genus (Illiger, 1811) [145,146]. These findings have collectively suggested the positive value of *s* of the CENPB-like box. The preservation of both the conserved and variable domains across 18 different populations suggested that cen-satDNA was evolutionarily constrained in long-tailed and rhesus macaques [31,41,147,148,149]. Differential rates of substitution in cen-satDNA could have resulted from the interaction of DNA-binding proteins with satellite DNA. Immunofluorescence colocalization using polyclonal anti-poly (ADP-ribose) polymerase (PARP) antibody, anti-5-methylcytosine (5meC) antibody, remodeling and spacing factor (RSF) complex (Rsf-1), or SNF2h antibody are required to confirm this functional possibility [150,151,152].

### 4.2. Nonrandom Cen-satDNA Sequences in Long-Tailed and Rhesus Macaque Chromosomes

Our chromosomal analysis showed that all cen-satDNA subfamilies were mapped to the centromeric region in all chromosomes, except for the Y chromosome, suggesting their common evolution and expansion in the genome. Similar cases of different cen-satDNA sequences between autosome/X and Y chromosomes were observed in alpha-satellite families in sun-tailed monkeys (*Cercopithecus solatus*, Harrison 1988) [59,153]. The absence of cen-satDNA on the Y chromosome in long-tailed and rhesus macaques might be explained by chromosomal rearrangement, such as unequal crossing over between the X and Y chromosomes, resulting in its reduction or almost absence in the Y. The lack of conservation and prevalence of satDNA sequences in the neocentromeres of the genome have raised questions whether any specific satellite sequences are required for function [5,13]. In addition, it has been reported that the deleterious effect of an increase in one satellite DNA group is mitigated by a decrease in a different group on the Y chromosome [154]. Meiotic drive has been proposed as the likely explanation for the salutatory divergence of satDNA sequences and the excess nonsynonymous divergence of several centromere proteins, some of which interact directly with the Y, together with the fitness benefits of maintaining an optimal genome size [21,138,139,155,156]. Alternatively, it has been proposed that the Y chromosome is probably excluded from recombination events with nonhomologous chromosomes [157]. Such changes might contribute to the significant inter- and intraspecific heterogeneity of the genome size observed in insects [158,159]. In great apes, the nonrandom distribution of various satDNA subfamilies of SatIII DNA has been observed on different chromosomes [160]. Several satDNA subfamilies are present on the human Y chromosome, more than on the human acrocentric chromosomes or the nonhuman primate Y chromosomes, suggesting that the reduction in the number of satDNAs in less ancient primates is due to the loss of the number of repeats in some subfamilies [161,162,163]. However, in other studies, these satDNA subfamilies were undetectable by FISH due to the low copy number or large sequence divergence on the centromeric region of Y, and the nature of highly repetitive sequences obstructing genome assembly [164].

Considering the distribution of cen-satDNA sequences on autosomes and X chromosomes, our analysis of the arrangement of cen-satDNA on chromosomes 3 and 8 of long-tailed macaque and chromosomes 18 and 19 of rhesus macaque revealed the presence of distinct satellite arrays forming structural complexes or possible HORs that might have resulted from centromere repositioning, which is known to recruit a large block of alpha-satDNA leading to rearrangement [165]. This was consistent with the presence of at least nine repositioned centromeres in the macaque lineage [166]. The chromosome-specific distribution of cen-satDNA might ensure faithful chromosomal transmission or prevent deleterious rearrangements, as lengthy satellite blocks might be more prone to unequal crossing over or ectopic recombination [154]. Similar patterns in gibbons and New World monkeys were also associated with the existence of chromosome-specific centromeric sequences [167].

### 4.3. Cen-satDNA in Long-Tailed and Rhesus Macaque Populations

The mean satellite abundance in a population is expected to approximate an equilibrium determined by the mutation rate, the degree of selective constraint, potentially positive selection, and population size. A high *F*_ST_ value suggests a low rate of genetic exchange between populations. Most variations in cen-satDNA sequences were observed among individuals within populations, probably resulting from the extensive genetic differentiation between populations. In addition, both meiotic drive and segregation distortion also promote strong population-specific patterns [154]. Similar patterns of *H_o_* and *H_e_* among populations together with STRUCTURE and LSI analyses indicated no geographical bias in the amplification of cen-satDNA subfamilies in any population, suggesting that cen-satDNA divergence at the population level did not identify population-specific mutations or other population-specific features of satDNA sequences. These findings opposed hypothesis (ii) regarding there being no bias of cen-satDNA diversity in any population. The population structure inferred from overall satellite quantities did not recapitulate the expected population relationships. Moreover, the observed admixture patterns that are lacking in population differentiation suggested individual-specific genetic differentiation among satellite sequences.

The demographically isolated macaque populations in Thailand have resulted from the integrated effects of the expanding human population size and human activities [168,169]. Consequently, the inbreeding process in populations with multiple satDNA subfamilies might drive the homogenization of satellite arrays in different populations. Interestingly, both long-tailed and rhesus macaques can produce hybrids with similar genomic profiles. This has suggested that differences in cen-satDNA profiles might not have resulted from hybrid incompatibility between the two species, leading to a lack of chromosomal segregation bias in each species or their hybrids. In these scenarios, chromosomes with a particular k-mer abundance or a composition with better fitness have a segregation advantage in the population. If these cen-satDNA sequences have pleiotropic deleterious effects, the fixation of a suppressor can quickly purge these sequences from populations [154]. As such, negative selection, that is, purging repetitive DNA, might have occurred in these populations [170].

## 5. Conclusions

This study investigated the genetic variability and genomic organization of cen-satDNA in two biomedically important primate model species, the long-tailed and rhesus macaques, at the population scale. Several chromosomal approaches have provided unprecedented insights into the diversity and organization of cen-satDNA at the individual, populational, and species levels. The process of diversification was apparent at all levels, indicating the increased occurrence of satellite turnover leading to the concerted evolution of cen-satDNA in both macaque species. This study attempted to unveil the complex evolutionary mechanisms that result in the expansion and homogenization of centromeric repeats. The recent emergence of long-read sequencing technologies (Oxford nanopore and PacBio sequencing) and complete centromere assemblies will provide more insights toward understanding the true nature of primate centromere evolution. Future research should address the biological consequences of sequence variation and the diversity of cen-satDNA on the adaptive evolution of primates. Our study provided evidence for the significant variation of satellite DNA profiles at the population level; however, further research is required to explore the potential influence of satellite DNA dynamics underlying the evolutionary mechanisms and functions involved in the process of speciation.

## Figures and Tables

**Table 1:** Sequence analysis of candidate clones and conserved motifs of satDNA sequences found in all sequence units.

Sequence Lengths (bp)	GC Content	Motif Sequences
170–174	36.8–41.5%, (average of 39.5%)	Motif 1: CTCACAGAGTTAC Motif 2: CTTTCTGAGAAACT

**Table 2:** Estimated neutrality tests in macaque populations using centromeric satDNA sequences.

Population	Tajima’s *D* Statistic	Fu and Li’s *D* Test	Fu and Li’s *F* Test
BPN	1.34 ^ns^	0.343 ^ns^	0.872 ^ns^
BSS	2.69 ^ns^	0.450 ^ns^	1.547 ^ns^
KN	1.17 ^ns^	−0.603 ^ns^	0.200 ^ns^
KNG	3.52 ^ns^	−0.842 ^ns^	0.918 ^ns^
KNKTK	2.72 ^ns^	0.085 ^ns^	1.218 ^ns^
KSP	1.15 ^ns^	−0.397 ^ns^	0.284 ^ns^
MFRC	−1.01 ^ns^	0.398 ^ns^	0.519 ^ns^
SSD	3.87 ^ns^	-0.549 ^ns^	1.221 ^ns^
SY	2.36 ^ns^	0.885 ^ns^	1.580 ^ns^
WHM	1.57 ^ns^	0.235 ^ns^	0.948 ^ns^
WKH	0.82 ^ns^	−0.289 ^ns^	0.228 ^ns^
WKT	4.04 ^ns^	0.214 ^ns^	1.468 ^ns^
WPN	N/A	N/A	N/A
WPT	1.98 ^ns^	0.113 ^ns^	0.942 ^ns^
WSK	0.88 ^ns^	−0.285 ^ns^	0.284 ^ns^
WTM	1.05 ^ns^	−0.153 ^ns^	0.494 ^ns^
WTPMH	3.03 ^ns^	0.350 ^ns^	1.629 ^ns^
WTT	1.45 ^ns^	−0.024 ^ns^	0.672 ^ns^

ns = not significant. N/A = not available.

**Table 3:** Genetic diversity of macaque populations estimated using centromeric satDNA sequences. N: number of samples, *N_a_*: no. of different alleles, *N_e_*: no. of effective alleles, *I*: Shannon’s information index, *He*: expected heterozygosity, *Ho*: observed heterozygosity, *uHe*: unbiased expected heterozygosity, *F*: fixation index.

Population		N	*Na*	*Ne*	*I*	*Ho*	*He*	*uHe*	*F*
BPN	Mean	17	0.836	0.778	0.128	0.149	0.088	0.111	−0.597
	S.E.		0.067	0.061	0.022	0.028	0.016	0.021	0.031
BSS	Mean	40	1.008	0.921	0.197	0.198	0.136	0.170	−0.383
	S.E.		0.072	0.065	0.026	0.031	0.018	0.024	0.058
KN	Mean	18	0.820	0.714	0.134	0.139	0.089	0.105	−0.445
	S.E.		0.072	0.061	0.021	0.025	0.015	0.018	0.032
KNG	Mean	19	0.758	0.691	0.120	0.133	0.081	0.099	−0.535
	S.E.		0.069	0.061	0.021	0.026	0.015	0.019	0.031
KNKTK	Mean	20	0.875	0.847	0.162	0.213	0.115	0.162	−0.801
	S.E.		0.069	0.067	0.025	0.034	0.018	0.027	0.026
KSP	Mean	21	1.063	0.915	0.188	0.190	0.125	0.152	−0.417
	S.E.		0.072	0.059	0.024	0.027	0.016	0.021	0.028
MFRC	Mean	10	0.523	0.512	0.126	0.172	0.090	0.122	−0.891
	S.E.		0.070	0.068	0.023	0.033	0.017	0.023	0.021
SSD	Mean	20	0.852	0.788	0.174	0.217	0.121	0.189	−0.693
	S.E.		0.074	0.068	0.025	0.034	0.018	0.030	0.033
SY	Mean	14	0.883	0.804	0.123	0.137	0.083	0.106	−0.521
	S.E.		0.066	0.058	0.021	0.027	0.015	0.020	0.033
WHM	Mean	20	0.914	0.824	0.150	0.138	0.102	0.130	−0.293
	S.E.		0.070	0.062	0.023	0.027	0.016	0.022	0.057
WKH	Mean	19	0.875	0.779	0.132	0.139	0.088	0.109	−0.459
	S.E.		0.069	0.059	0.021	0.026	0.015	0.020	0.030
WKT	Mean	21	0.875	0.780	0.177	0.197	0.121	0.157	−0.505
	S.E.		0.076	0.067	0.024	0.031	0.017	0.024	0.045
WPN	Mean	6	0.727	0.715	0.110	0.140	0.078	0.125	−0.767
	S.E.		0.064	0.063	0.022	0.030	0.016	0.026	0.042
WPT	Mean	40	1.094	0.978	0.214	0.216	0.146	0.202	−0.377
	S.E.		0.072	0.064	0.026	0.032	0.018	0.028	0.056
WSK	Mean	20	1.070	1.024	0.183	0.228	0.128	0.188	−0.720
	S.E.		0.063	0.059	0.026	0.034	0.018	0.029	0.028
WTM	Mean	20	0.766	0.729	0.125	0.160	0.088	0.110	−0.732
	S.E.		0.068	0.064	0.023	0.031	0.016	0.022	0.031
WTPMH	Mean	33	1.695	1.428	0.359	0.382	0.241	0.278	−0.420
	S.E.		0.041	0.037	0.025	0.035	0.018	0.023	0.045
WTT	Mean	20	1.648	1.345	0.325	0.323	0.214	0.240	−0.373
	S.E.		0.045	0.036	0.024	0.030	0.017	0.020	0.031
Total	Mean	377	0.960	0.865	0.174	0.193	0.119	0.153	−0.510
	S.E.		0.017	0.015	0.006	0.007	0.004	0.006	0.010

**Table 4:** Analysis of molecular variance (AMOVA) in the 18 total populations.

Source	df	Sum of Squares	% Variation	*F*-Statistics
among populations	17	821.495	4%	*F_ST_* = 0.038 *
among individuals	359	8864.168	67%	*F_IS_* = 0.701 *
within individuals	377	1634.500	29%	*F_IT_* = 0.713 *
total	753	11320.163	100%	

* *p* < 0.001.

## Data Availability

The full dataset and metadata from this study are available from the Dryad Digital Repository Dataset at https://doi.org/10.5061/dryad.w3r2280sw (accessed on 1 December 2021).

## Supplementary Materials

The following supporting information can be downloaded at: https://www.mdpi.com/article/10.3390/cells11121953/s1, Figure S1: Secondary structure of 5 units within the clone insert using the Mfold web server Zuker (2003).; Figure S2: k-mer-based characterization and evolution of cen-satDNA families. (a) MSA visualization showing the region of conservation and significant variations among satellite families. (b) Phylogenetic relationship among identified families. (c) TAREAN graphics of 10 predicted subfamilies showing distinct topology and patterns of k-mer abundance.; Figure S3: Genomic organization of complex cen-satDNA tandem arrays in the centromeric regions in the genome of *M**. mulatta*, visualized as heatmaps with StainedGlass for (a) chromosome 18 and (b) chromosome 19. (c) Histogram shows the variable number of alignments compared with the percentage identity in these regions.; Figure S4: Genomic organization of complex cen-satDNA tandem arrays in the centromeric regions in the genome of *M**. fascicularis*, visualized as heatmaps with StainedGlass for (a) chromosome 18 and (b) chromosome 19. (c) Histogram shows the variable number of alignments compared with the percentage identity in these regions.; Figure S5: Genomic variability of cen-satDNA across different primates. Boxplot shows the distribution of sequence length for alignments (restricted to the top 100 hits) reported for each species.; Figure S6: Population scale phylogenetic tree of macaques inferred from cen-satDNA SNPs detected in all individuals. Each color corresponds to a single population (consistent with color legends in Figure 4). BPN: Baan Pak Nam, Suratthani; BSS: Baan Sang School, Buengkarn; KN: Khao Nor, Nakhonsawan; KNG: Khao Ngu, Ratchaburi; KNKTK: Khao Noi Khao Tangkuan, Songkhla; KSP: Kosumpi, Mahasarakham; MFRC: Mangrove Forest Research Center, Ranong; SSD: Somdet Phra Sinagarindra Park, Chumphon; SY: Sai Yok, Kanchanaburi; WHM: Wat Haad Moon, Pichit; WKH: Wat Kuha Pimuk, Yala; WKT: Wat Khao Thamon, Petchaburi; WPN: Wat Pak Nam, Ranong; WPT: Wat Pattanajit, Nakhon Phanom; WSK: Wat Suwan Khuha, Pang-nga; WTM: Watthammasala, Nakhon Pathom; WTPMH: Wat Tham Pa Mak Ho, Loei; and WTT: Wat Tham Thep Ban Dan, Phetchabun. Table S1: Sampled populations of macaques at location sites in Thailand; Table S2: k-mer-based characterization of subfamilies in macaque cen-satDNA; Table S3: Chromosomal localization and genomic organization of cen-satDNA with respective alignments in macaque genomes; Supplementary Dataset S1: Genomic features of cen-satDNA-assembled contigs in macaque populations; Supplementary Dataset S2: Comparative alignments of cen-satDNA across different primate species; Supplementary Dataset S3: Comparison of Ho and He between 18 populations.
