# Supplementary material for: Population Scale Analysis of Centromeric Satellite DNA Reveals Highly Dynamic Evolutionary Patterns and Genomic Organization in Long-Tailed and Rhesus Macaques
Source: Cells. 2022 Jun 17;11(12):1953. doi: 10.3390/cells11121953 (PMC9221937; doi:10.3390/cells11121953)
Supplement: Supplementary file 1 [file cells-11-01953-s001.zip › cells-1756620-supplementary figures.pdf]

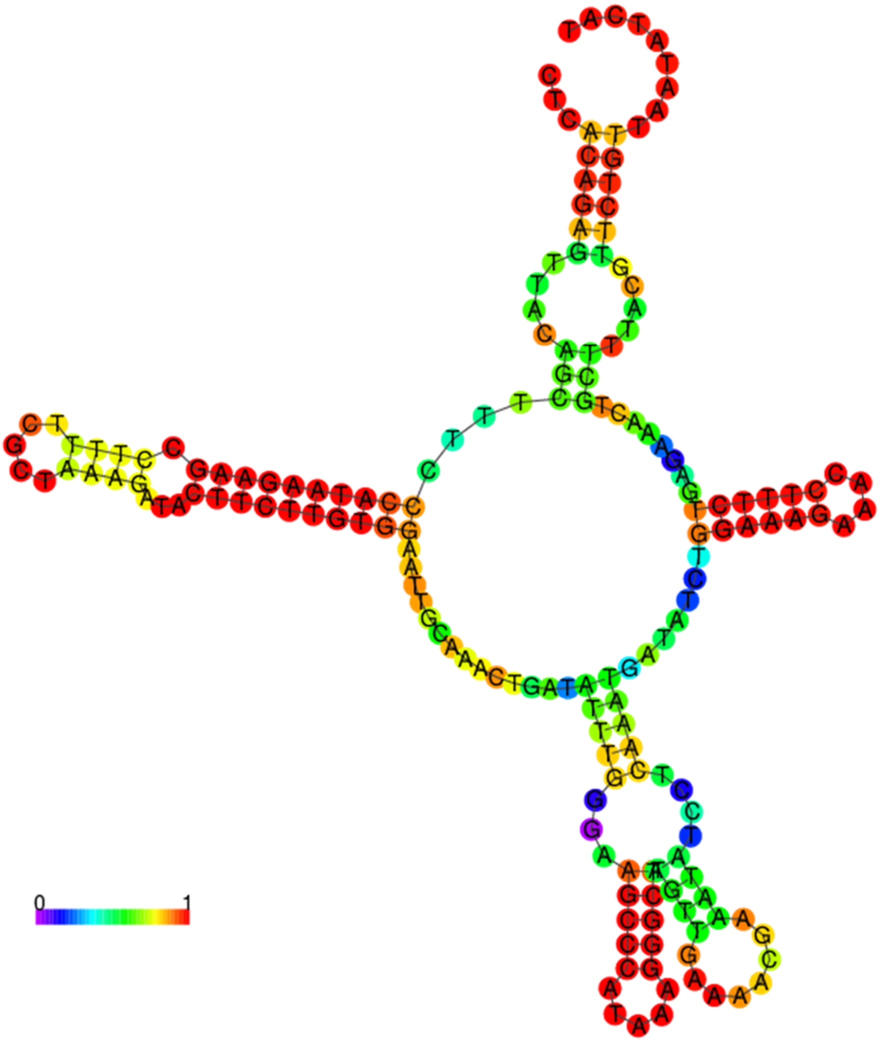


**Supplementary Figure S1.** Secondary structure of 5 units within the clone insert using the Mfold web server Zuker (2003).

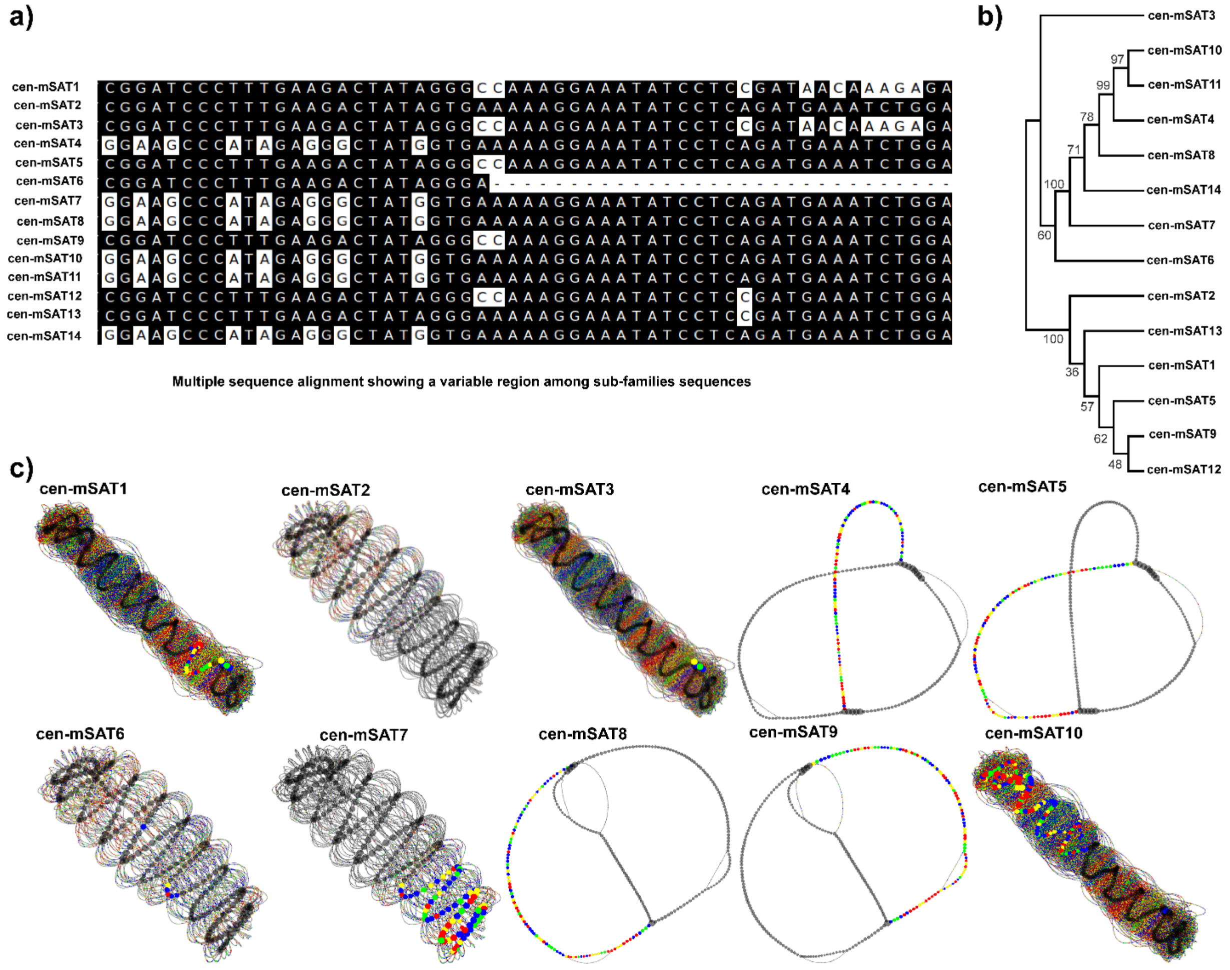


**Supplementary Figure S2.** k-mer-based characterization and evolution of cen-satDNA families. a) MSA visualization showing the region of conservation and significant variations among satellite families. b) Phylogenetic relationship among identified families. c) TAREAN graphics of 10 predicted subfamilies showing distinct topology and patterns of k-mer abundance.

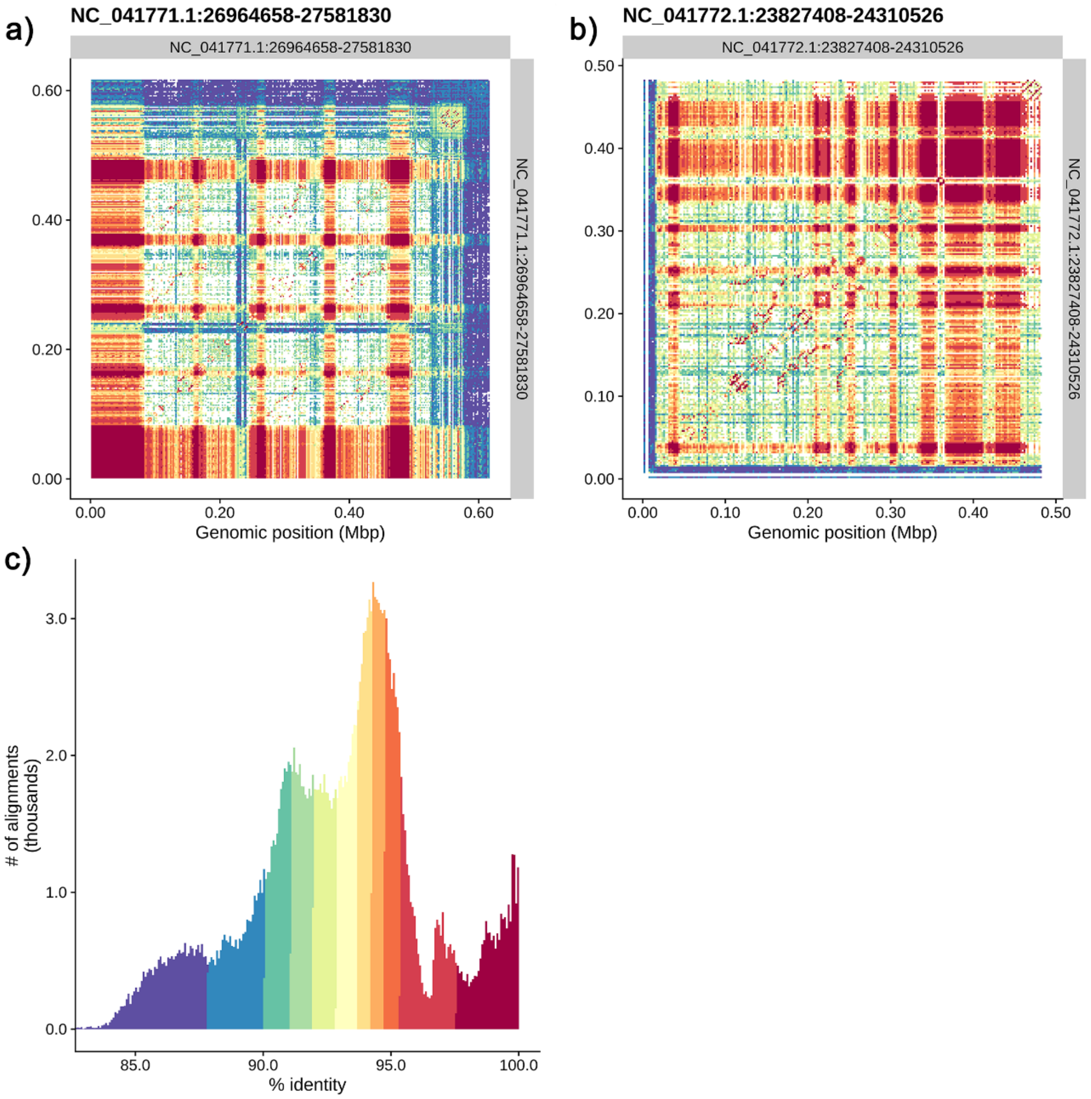


**Supplementary Figure S3.** Genomic organization of complex cen-satDNA tandem arrays in the centromeric regions in the genome of *M. mulatta*, visualized as heatmaps with StainedGlass for a) chromosome 18 and b) chromosome 19.c) Histogram shows the variable number of alignments compared with the percentage identity in these regions.

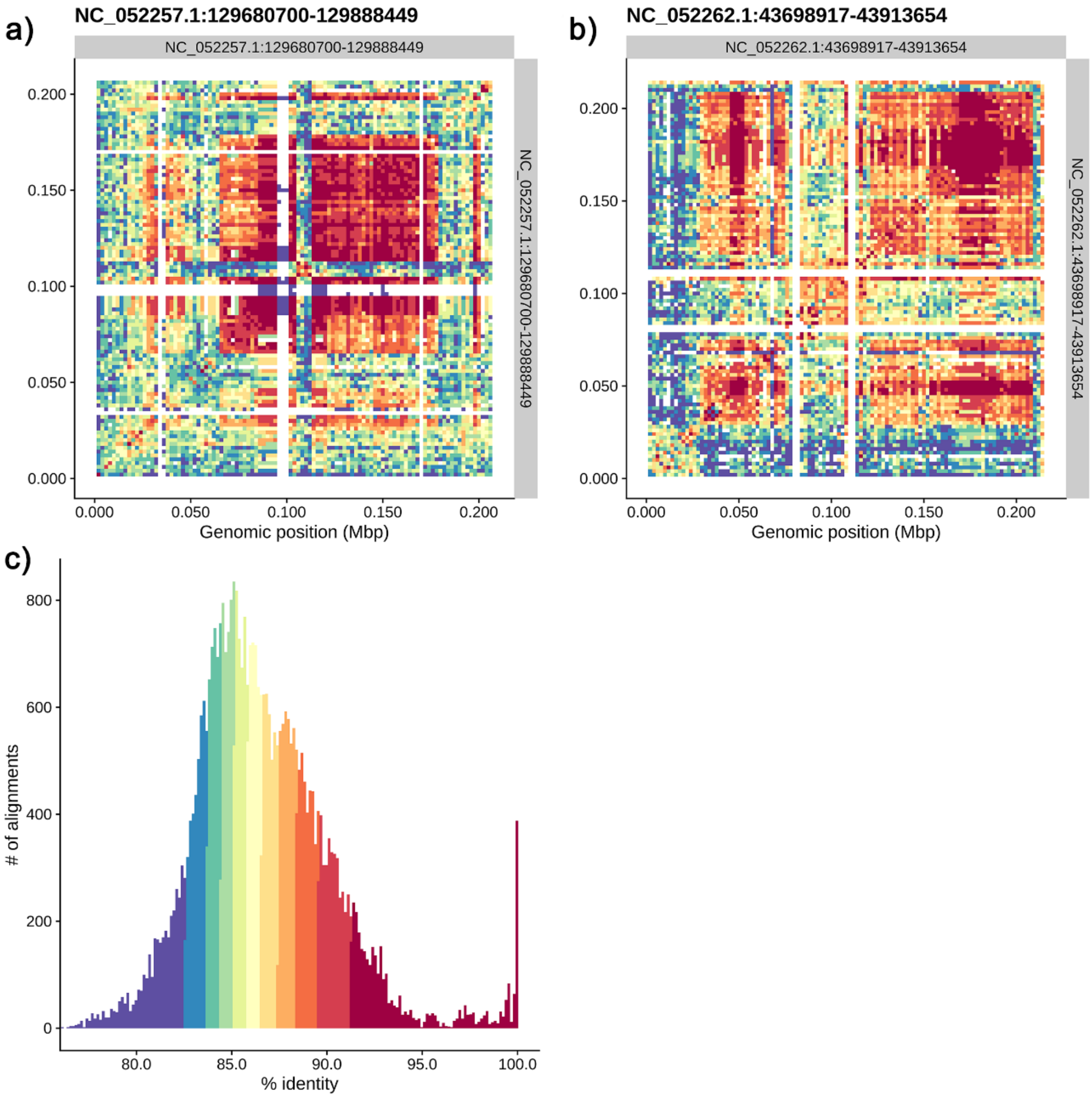


**Supplementary Figure S4.** Genomic organization of complex cen-satDNA tandem arrays in the centromeric regions in the genome of *M. fascicularis*, visualized as heatmaps with StainedGlass for a) chromosome 18 and b) chromosome 19. c) Histogram shows the variable number of alignments compared with the percentage identity in these regions.

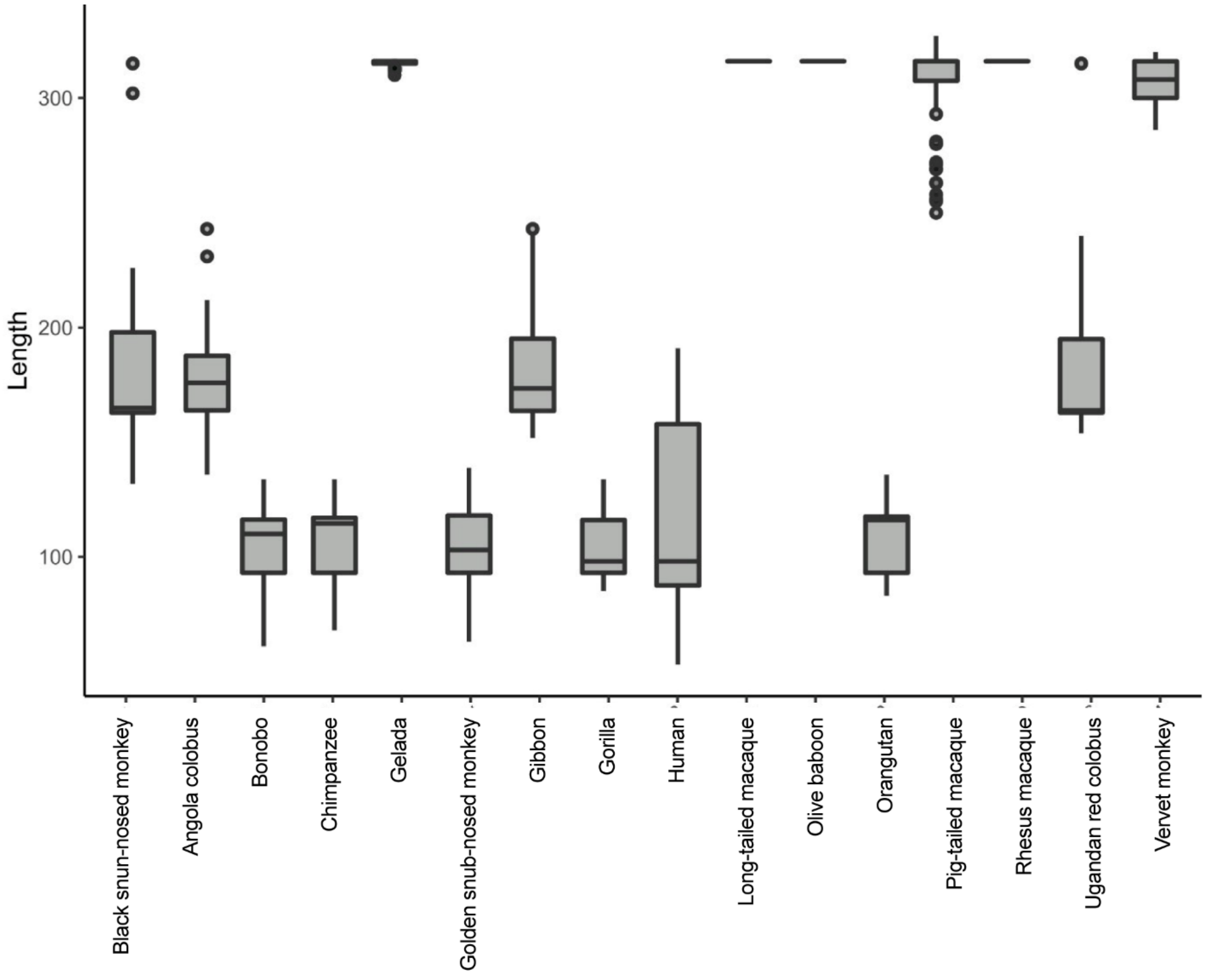


**Supplementary Figure S5.** Genomic variability of cen-satDNA across different primates. Boxplot shows the distribution of sequence length for alignments (restricted to the top 100 hits) reported for each species.

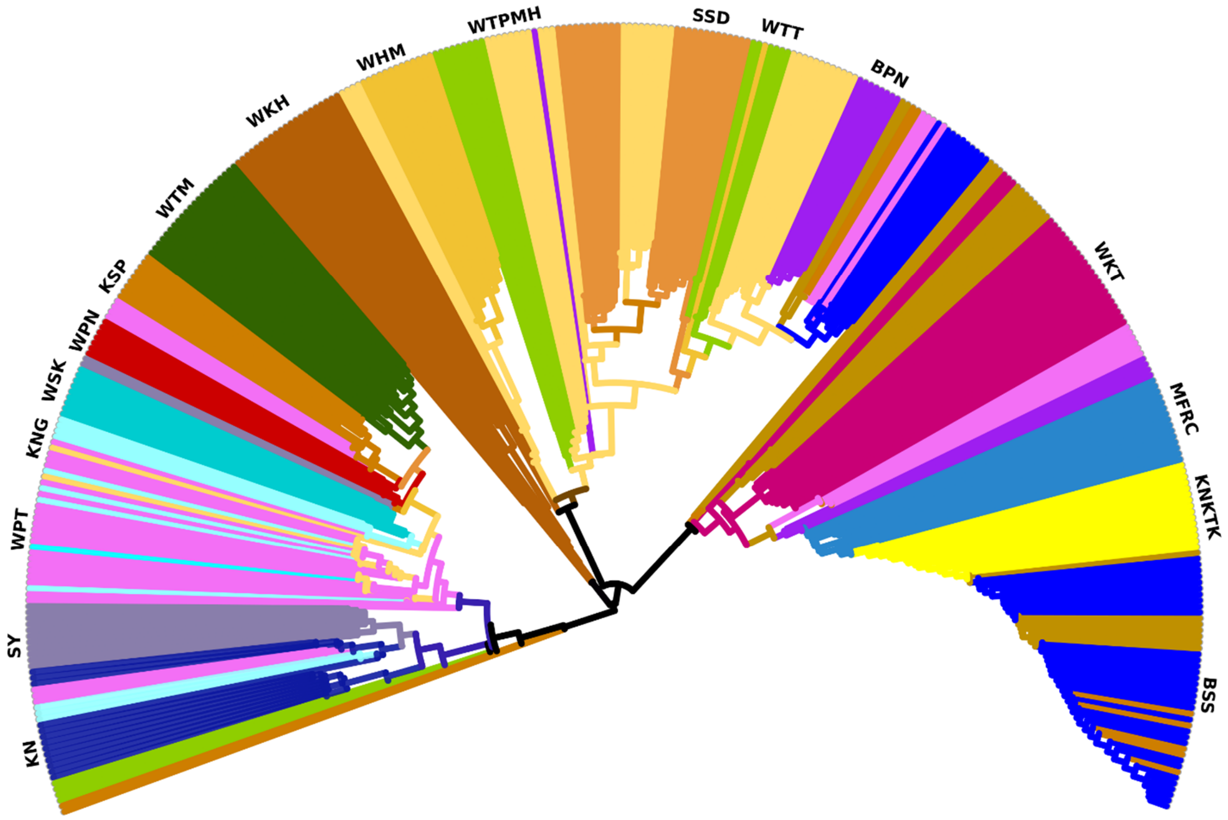


**Supplementary Figure S6.** Population scale phylogenetic tree of macaques inferred from cen-satDNA SNPs detected in all individuals. Each color corresponds to a single population (consistent with color legends in Figure 4). BPN; Baan Pak Nam, Suratthani, BSS; Baan Sang School, Buengkarn, KN; Khao Nor, Nakhonsawan, KNG; Khao Ngu, Ratchaburi, KNKTK; Khao Noi Khao Tangkuan, Songkhla, KSP; Kosumpi, Mahasarakham, MFRC; Mangrove Forest Research Center, Ranong, SSD; Somdet Phra Sinagarindra Park, Chumphon, SY; Sai Yok, Kanchanaburi, WHM; Wat Haad Moon, Pichit, WKH; Wat Kuha Pimuk, Yala, WKT; Wat Khao Thamon, Petchaburi, WPN; Wat Pak Nam, Ranong, WPT; Wat Pattanajit, Nakhon Phanom, WSK; Wat Suwan Khuha, Pang-nga, WTM; Watthammasala , Nakhon Pathom, WTPMH; Wat Tham Pa Mak Ho, Loei, and WTT; Wat Tham Thep Ban Dan, Phetchabun.
