# Supplementary material for: Population Scale Analysis of Centromeric Satellite DNA Reveals Highly Dynamic Evolutionary Patterns and Genomic Organization in Long-Tailed and Rhesus Macaques
Source: Cells. 2022 Jun 17;11(12):1953. doi: 10.3390/cells11121953 (PMC9221937; doi:10.3390/cells11121953)
Supplement: Supplementary file 1 [file cells-11-01953-s001.zip › cells-1756620-supplementary tables.pdf]

Table S1. Sampled populations of macaques at location sites in Thailand.

Abbreviation/Code	Number of specimen	Location	Geographic coordinates	Species
BPN	17	Baan Pak Nam, Suratthani	8.999800, 98.817565	*Macaca fascicularis fascicularis*
BSS	40	Baan Sang School, Buengkarn	17.859684, 103.959704	*Macaca mulatta*
KN	18	Khao Nor, Nakhonsawan	15.944844, 99.876868	*Macaca fascicularis fascicularis*
KNG	19	Khao Ngu, Ratchaburi	13.569441, 99.774925	*Macaca fascicularis fascicularis*
KNKTK	20	Khao Noi Khao Tangkuan, Songkhla	7.211619, 100.593992	*Macaca fascicularis fascicularis*
KSP	21	Kosumpi, Mahasarakham	16.253749, 103.067346	*Macaca fascicularis fascicularis*
MFRC	10	Mangrove Forest Research Center, Ranong	9.875440, 98.604602	*Macaca fascicularis aurea*
SSD	20	Somdet Phra Sinagarindra Park, Chumphon	9.947496, 99.039400	*Macaca fascicularis fascicularis*
SY	14	Sai Yok, Kanchanaburi	14.450672, 98.850287	*Macaca fascicularis fascicularis*
WHM	20	Wat Haad Moon, Pichit	16.505553, 100.281847	*Macaca fascicularis fascicularis*
WKH	19	Wat Kuha Pimuk, Yala	6.527541, 101.224451	*Macaca fascicularis fascicularis*
WKT	21	Wat Khao Thamon, Petchaburi	13.046734, 99.951231	*Macaca fascicularis fascicularis*
WPN	6	Wat Pak Nam, Ranong	9.953746, 98.589608	*Macaca fascicularis aurea*
WPT	40	Wat Pattanajit, Nakhon Phanom	17.083981, 104.511482	*Macaca mulatta*
WSK	20	Wat Suwan Khuha, Pang-nga	8.428850, 98.471612	*Macaca fascicularis fascicularis*
WTM	20	Watthammasala , Nakhon Pathom	13.812083, 100.114738	*Macaca fascicularis fascicularis*
WTPMH	33	Wat Tham Pa Mak Ho, Loei	17.233897, 101.780720	*Macaca mulatta*
WTT	20	Wat Tham Thep Ban Dan, Phetchabun	15.748736, 101.035112	*Macaca fascicularis fascicularis*

Table S2. Kmer-based characterization of subfamilies in macaque cen-satDNA

Satellite Family	Kmer length (bp)	Variant index	K-mer coverage score	Predicted Monomers (bp)
cent-Msat1	27	1	0.55	172
cent-Msat2	23	2	0.54	171
cent-Msat3	27	3	0.51	343
cent-Msat4	11	2	0.47	172
cent-Msat5	11	1	0.45	171
cent-Msat6	23	5	0.44	343
cent-Msat7	23	3	0.42	172
cent-Msat8	15	2	0.41	172
cent-Msat9	15	1	0.39	171
cent-Msat10	27	2	0.37	172
cent-Msat11	19	2	0.36	172
cent-Msat12	19	1	0.33	171
cent-Msat13	23	1	0.24	169
cent-Msat14	23	4	0.24	173

Table S3. Chromosomal localization and genomic organization of cen-satDNA based on alignment in respective macaque genomes

**Rhesus macaque**				
**Chromosome NCBI ID**	**Chromosome name**	**Start position (bp)**	**End position (bp)**	**Number of cen-satDNA alignments**
NC_041754.1	Chromosome 1	87,650,676	87,771,732	1,269
NC_041755.1	Chromosome 2	65,732,492	65,815,243	260
NC_041755.1	Chromosome 2	66,984,563	67,579,764	4,452
NC_041756.1	Chromosome 3	55,318,685	55,321,036	2
NC_041756.1	Chromosome 3	55,650,778	55,869,407	1,703
NC_041757.1	Chromosome 4	49,410,049	49,571,840	1,708
NC_041757.1	Chromosome 4	50,646,191	50,719,328	1,185
NC_041758.1	Chromosome 5	48,529,732	48,549,734	29
NC_041758.1	Chromosome 5	82,067,638	82,346,004	3,022
NC_041758.1	Chromosome 5	83,346,005	83,429,258	380
NC_041759.1	Chromosome 6	46,432,987	46,617,448	562
NC_041759.1	Chromosome 6	47,617,455	47,875,507	4,123
NC_041760.1	Chromosome 7	59,764,658	59,885,848	361
NC_041760.1	Chromosome 7	60,885,863	60,985,042	801
NC_041761.1	Chromosome 8	44,846,708	45,193,714	3,817

NC_041761.1	Chromosome 8	46,193,803	46,237,201	599
NC_041762.1	Chromosome 9	1	35,502	156
NC_041762.1	Chromosome 9	38,632,896	38,807,112	3,010
NC_041762.1	Chromosome 9	41,530,338	41,628,824	328
NC_041763.1	Chromosome 10	37,811,900	37,901,665	722
NC_041764.1	Chromosome 11	36,400,064	36,929,713	5,353
NC_041764.1	Chromosome 11	37,929,773	37,996,966	987
NC_041764.1	Chromosome 11	38,290,579	38,316,450	12
NC_041764.1	Chromosome 11	98,387,203	98,387,508	15
NC_041765.1	Chromosome 12	23,463,668	2,3624,702	299
NC_041765.1	Chromosome 12	24,625,279	24,702,686	255
NC_041766.1	Chromosome 13	19,815,670	20,046,407	821
NC_041766.1	Chromosome 13	21,248,652	21,458,908	1,877
NC_041767.1	Chromosome 14	60,444,990	60,647,941	983
NC_041767.1	Chromosome 14	61,648,126	62,060,218	4,684
NC_041768.1	Chromosome 15	18,805,172	18,927,257	940
NC_041768.1	Chromosome 15	19,927,278	20,001,249	828
NC_041769.1	Chromosome 16	21,831,078	22,037,084	1,815
NC_041770.1	Chromosome 17	42,785,447	42,857,230	317

NC_041770.1	Chromosome 17	46,670,518	46,694,778	191
NC_041771.1	Chromosome 18	25,807,458	25,964,657	1,594
NC_041771.1	Chromosome 18	26,964,658	27,581,830	7,981
NC_041772.1	Chromosome 19	23,827,408	24,310,526	6,081
NC_041772.1	Chromosome 19	25,365,656	25,642,488	2,643
NC_041773.1	Chromosome 20	31,488,990	31,751,982	3,550
NC_041774.1	Chromosome 21	59,235,113	59,534,281	4,178
NC_041774.1	Chromosome X	59,787,912	59,788,447	4
NC_041774.1	Chromosome X	153,297,551	153,388,880	581
**Long-tailed macaque**				
NC_052255.1	Chromosome 1	86408389	86602023	335
NC_052256.1	Chromosome 2	127840778	127949959	76
NC_052256.1	Chromosome 2	128087615	128342568	256
NC_052257.1	Chromosome 3	129089879	129134705	244
NC_052257.1	Chromosome 3	129486163	129486503	1
NC_052257.1	Chromosome 3	129680700	129888449	1434
NC_052257.1	Chromosome 3	184885787	184904096	47
NC_052258.1	Chromosome 4	118605433	118635340	436
NC_052258.1	Chromosome 4	118990224	119014859	43

NC_052259.1	Chromosome 5	48502220	48523653	46
NC_052259.1	Chromosome 5	81981776	82034835	187
NC_052260.1	Chromosome 6	46302277	46479705	621
NC_052261.1	Chromosome 7	58088319	58212686	735
NC_052262.1	Chromosome 8	43698917	43913654	1634
NC_052263.1	Chromosome 9	39290774	39411677	420
NC_052263.1	Chromosome 9	41715178	41720722	50
NC_052264.1	Chromosome 10	6667	7176	3
NC_052265.1	Chromosome 11	35091449	35216613	263
NC_052265.1	Chromosome 11	95546558	95546863	15
NC_052267.1	Chromosome 13	19727737	19858343	186
NC_052268.1	Chromosome 14	65465608	65574565	158
NC_052269.1	Chromosome 15	93381979	93430059	235
NC_052270.1	Chromosome 16	55633514	55691499	1016
NC_052271.1	Chromosome 17	40444504	40449148	45
NC_052271.1	Chromosome 17	44801804	44824218	203
NC_052272.1	Chromosome 18	25767597	25918773	877
NC_052273.1	Chromosome 19	23772453	23822461	441
NC_052274.1	Chromosome 20	31185974	31217501	27

NC_052274.1	Chromosome 20	31912115	31957043	127
NC_052275.1	Chromosome X	58339519	58397258	106
NC_052275.1	Chromosome X	58650836	58651573	5
