# Supplementary figures and images for: Population Scale Analysis of Centromeric Satellite DNA Reveals Highly Dynamic Evolutionary Patterns and Genomic Organization in Long-Tailed and Rhesus Macaques
Source: Cells. 2022 Jun 17;11(12):1953. doi: 10.3390/cells11121953 (PMC9221937; doi:10.3390/cells11121953)

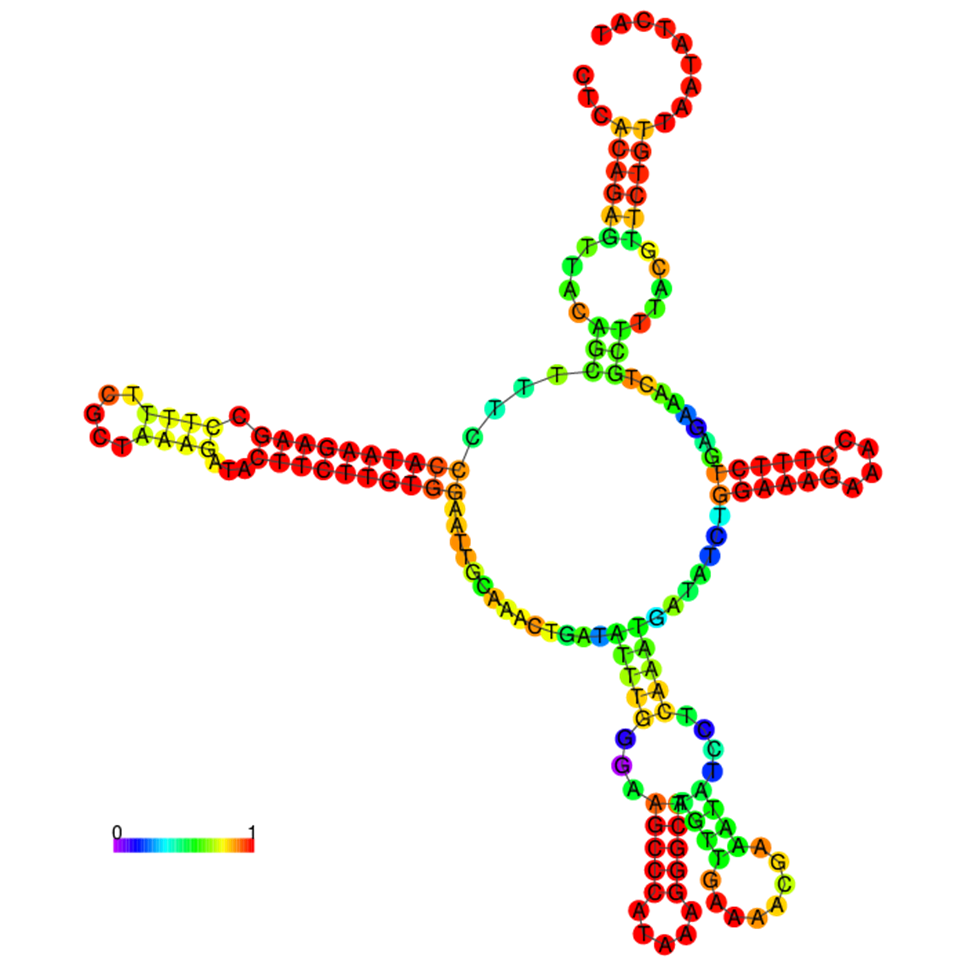

Supplement: Supplementary file 1 [file cells-11-01953-s001.zip › Figure S1 proof.tif]

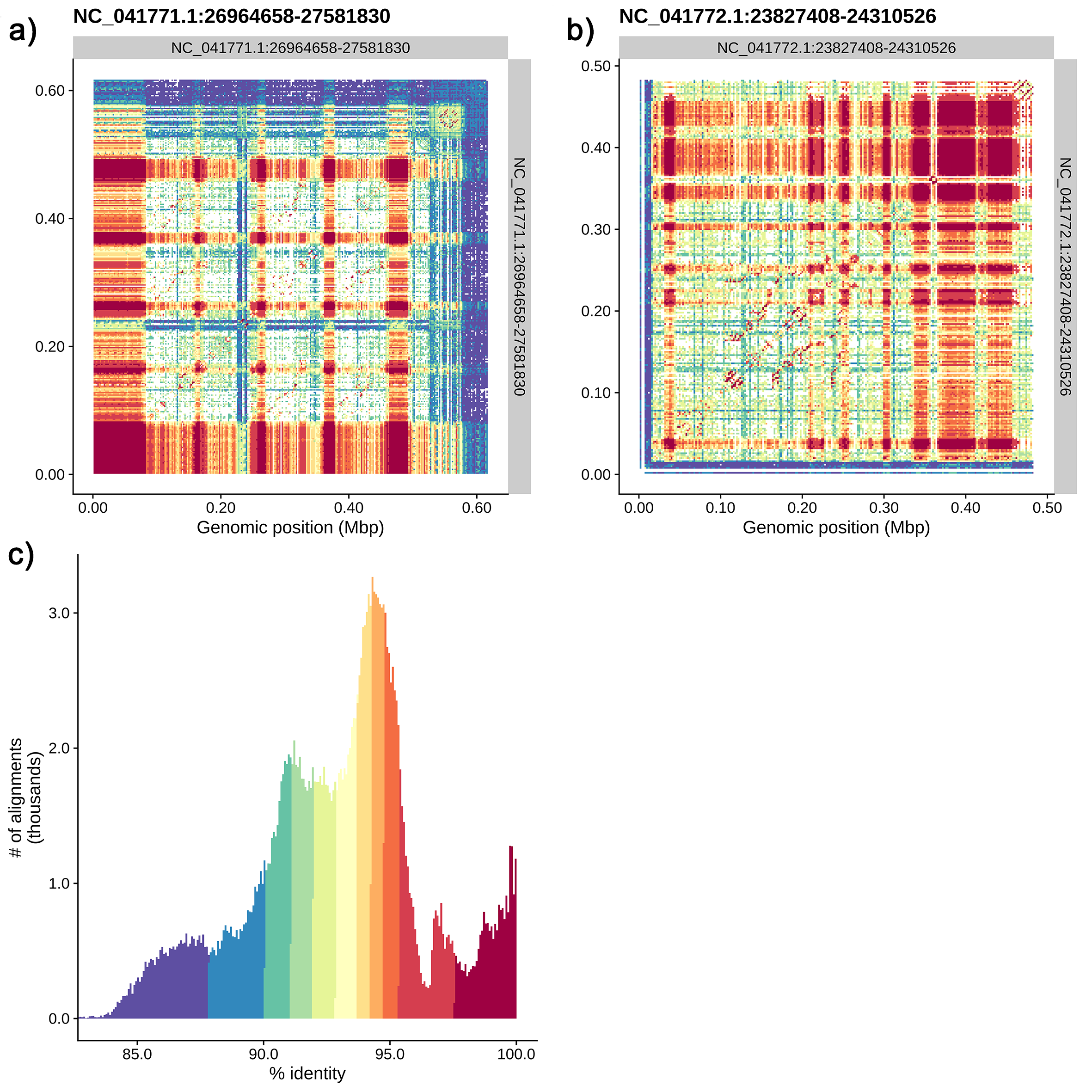

Supplement: Supplementary file 1 [file cells-11-01953-s001.zip › Figure S3 proof.tif]

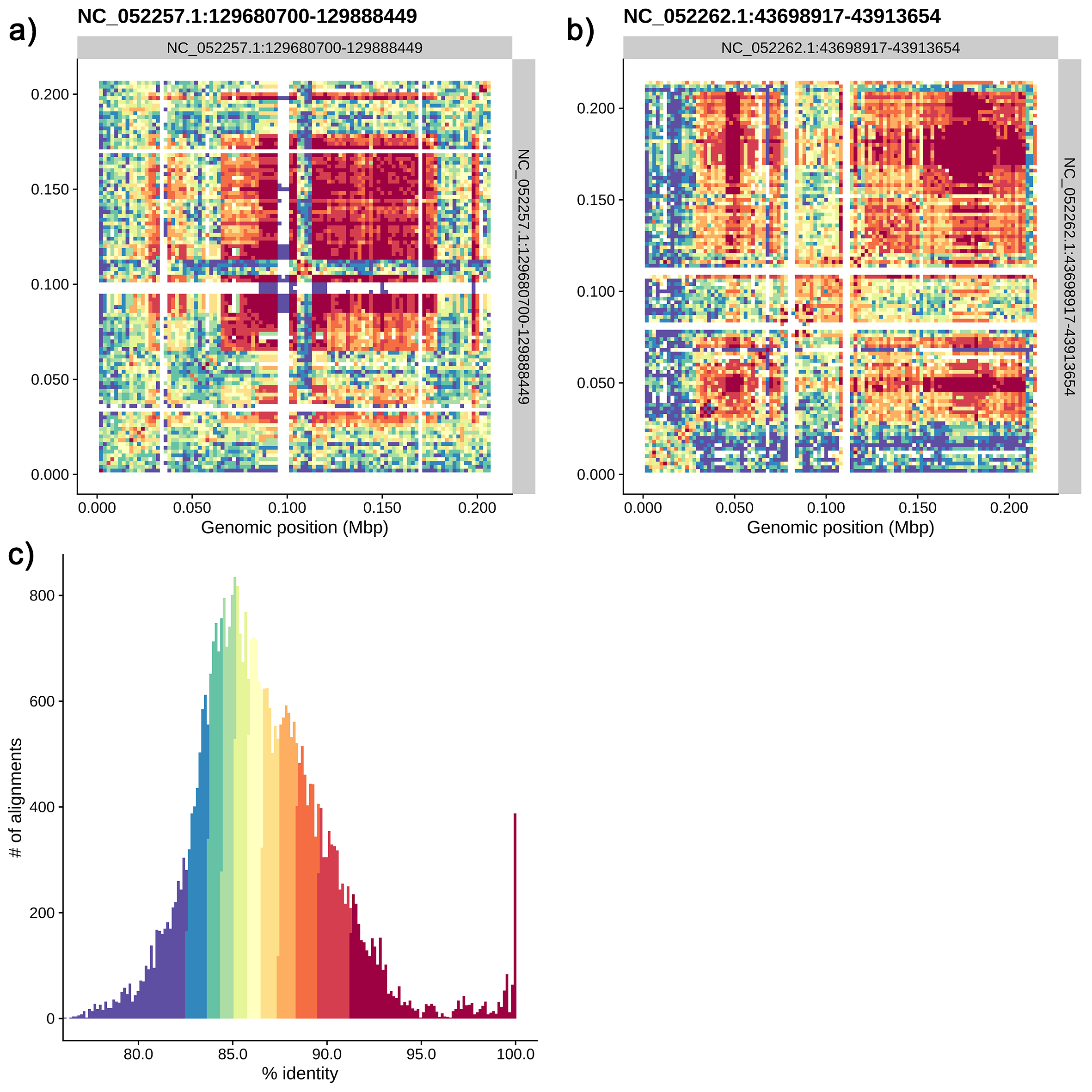

Supplement: Supplementary file 1 [file cells-11-01953-s001.zip › Figure S4 proof.tif]

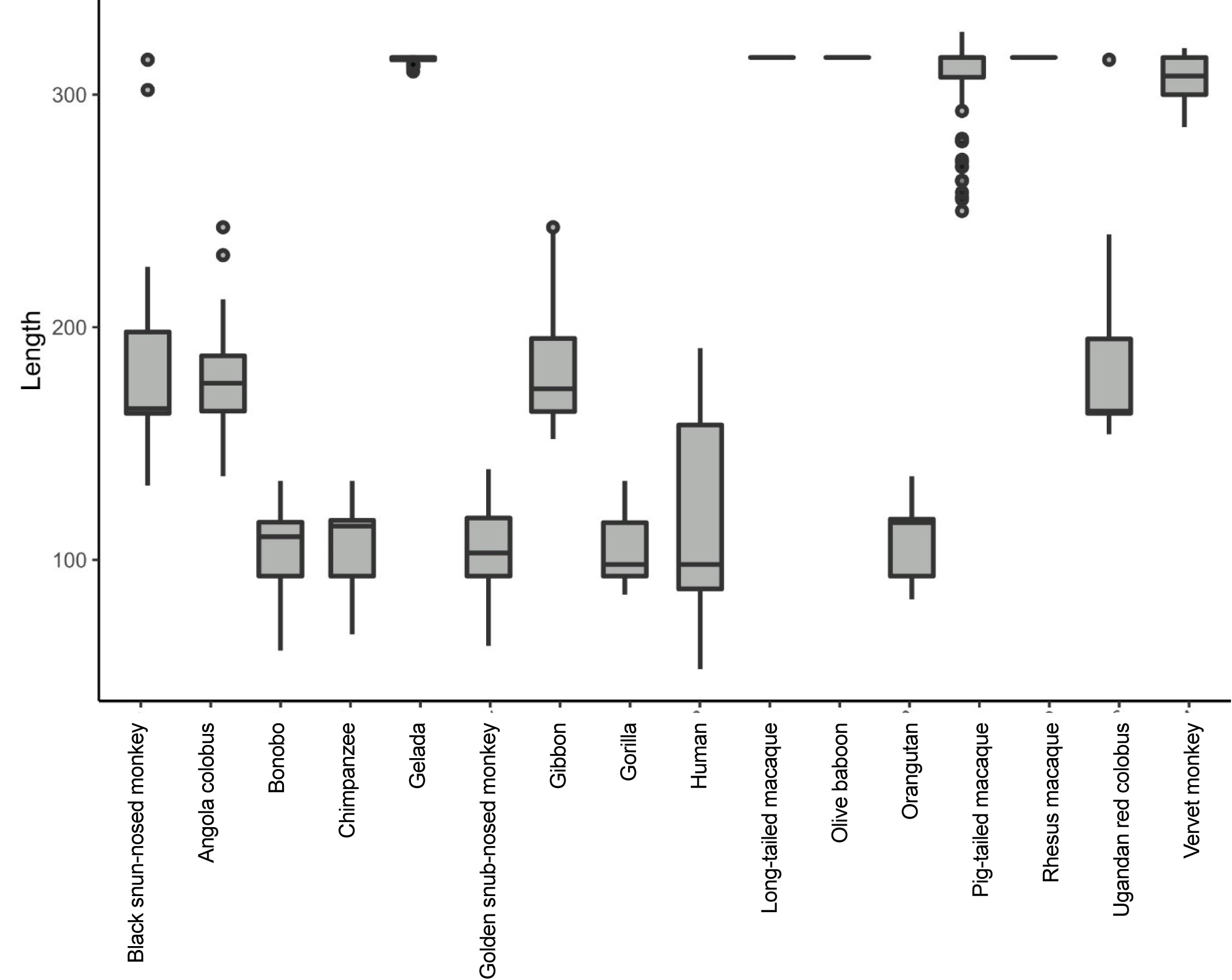

Supplement: Supplementary file 1 [file cells-11-01953-s001.zip › figure S5 proof.tif]

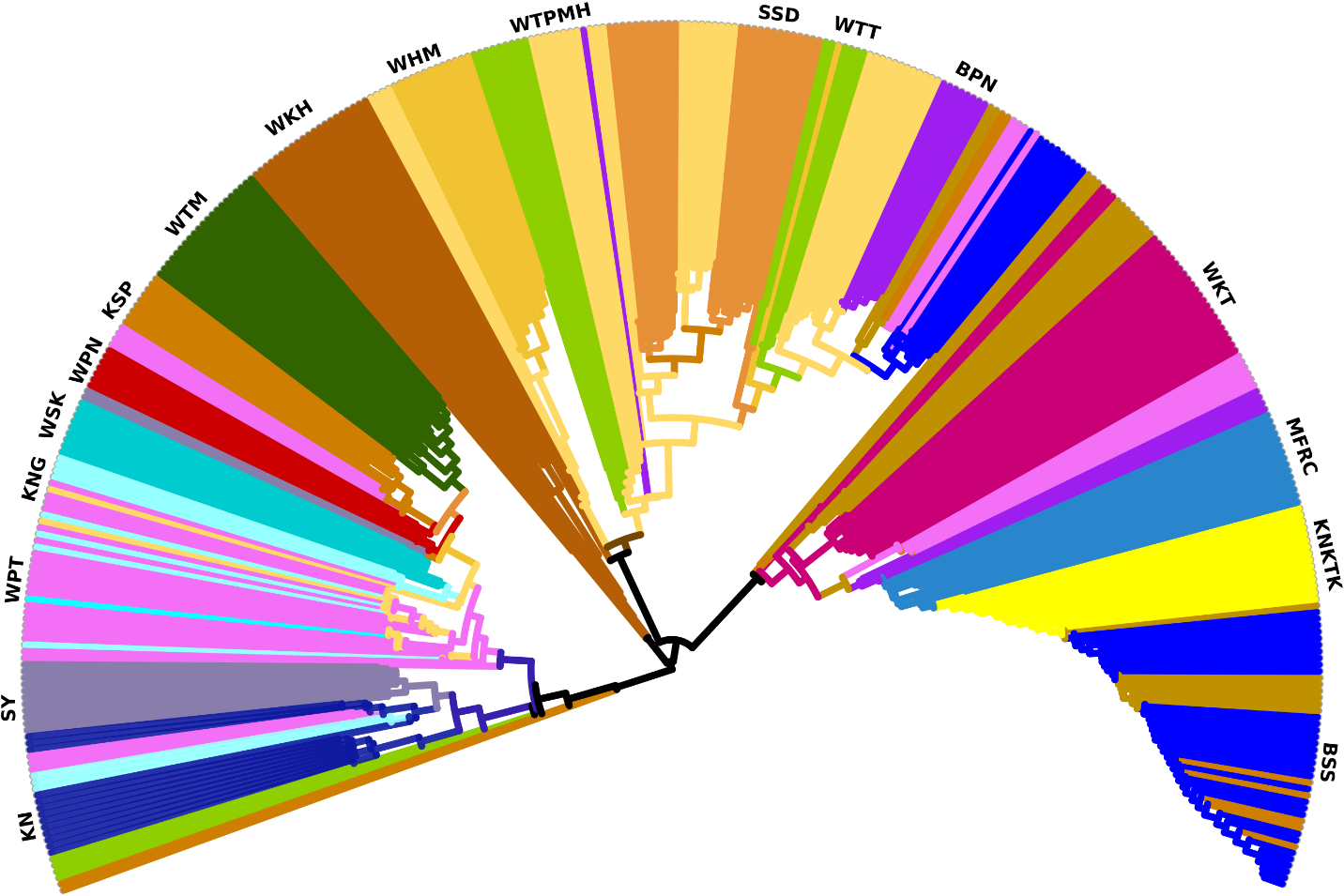

Supplement: Supplementary file 1 [file cells-11-01953-s001.zip › figure S6 proof.tif]
